# Network pharmacology integrated molecular docking reveals the bioactive components and potential targets of *Morinda officinalis*–*Lycium barbarum* coupled-herbs against oligoasthenozoospermia

**DOI:** 10.1038/s41598-020-80780-6

**Published:** 2021-01-26

**Authors:** Xue Bai, Yibo Tang, Qiang Li, Yafei Chen, Dan Liu, Guimin Liu, Xiaolei Fan, Ru Ma, Shuyan Wang, Lingru Li, Kailin Zhou, Yanfei Zheng, Zhenquan Liu

**Affiliations:** 1 School of Chinese Materia Medica, Beijing University of Chinese Medicine, Beijing, 100029 China; 2 School of Traditional Chinese Medicine, Beijing University of Chinese Medicine, Beijing, 100029 China; 3 National Institute of TCM Constitution and Preventive Medicine, Beijing University of Chinese Medicine, Beijing, 100029 China; 4 School of Humanities, Beijing University of Chinese Medicine, Beijing, 100029 China

**Keywords:** Male factor infertility, Urogenital diseases

## Abstract

Oligoasthenozoospermia (OA) is one of the most common types of male infertility affecting sperm count and sperm motility. Unfortunately, it is difficult for existing drugs to fundamentally improve the sperm quality of OA patients, because the pathological mechanism of OA has not been fully elucidated yet. *Morinda officinalis*–*Lycium barbarum* coupled-herbs (MOLBCH), as traditional Chinese Medicines, has been widely used for treating OA over thousands of years, but its molecular mechanism is still unclear. For this purpose, we adopted a comprehensive approach integrated network pharmacology and molecular docking to reveal the bioactive components and potential targets of MOLBCH against OA. The results showed that MOLBCH alleviated apoptosis, promoted male reproductive function, and reduced oxidant stress in the treatment of OA. Ohioensin-A, quercetin, beta-sitosterol and sitosterol were the key bioactive components. Androgen receptor (AR), Estrogen receptor (ESR1), Mitogen-activated protein kinase 3 (MAPK3), RAC-alpha serine/threonine-protein kinase (AKT1), Glyceraldehyde-3-phosphate dehydrogenase (GAPDH) were the core potential targets. PI3K/Akt signaling pathway, prostate cancer, AGE-RAGE signaling pathway in diabetic complications were the most representative pathways. Moreover, molecular docking was performed to validate the strong binding interactions between the obtained core components and targets. These observations provide deeper insight into the pathogenesis of OA and can be used to design new drugs and develop new therapeutic instructions to treat OA.

## Introduction

Infertility, defined as the inability to conceive after 12 months of regular and unprotected intercourse, is a complex multifactorial pathological condition affecting nearly 60–80 million couples worldwide^1^. Of all the infertile couples, male infertility contributes to approximately 50%^2^. Specifically, oligoasthenozoospermia (OA) is one of the most common types of male infertility^3^, which is defined as the total number or concentration of spermatozoa and percentage of progressively motile spermatozoa are below the lower reference limits (total number < 39 × 10^6^ per ejaculate; concentration < 15 × 10^6^ per ml; progressively motile < 32%)^4^. It is noted that recent studies have demonstrated sperm concentration and motility decreased for 72–90 days following Coronavirus Disease 2019 (COVID-19) infection, thus it is necessary to take a precaution measures towards to the treatment of OA^5–8^. According to the guidelines of the World Health Organization (WHO), OA is considered to be a condition that oligospermia and asthenozoospermia occur simultaneously^9^. Previous studies have shown that many factors impaired male reproductive function, which leads to OA accordingly, including varicocele, idiopathic, obstruction, cryptorchidism, immunologic, ejaculatory dysfunction, testicular failure, drug effects/radiation, endocrinology, and all others^10^. However, the molecular mechanism of OA has not been fully elucidated, and the current treatment has poor therapeutic effects and many limitations^11,12^. Assisted reproductive technology (ART) could improve the pregnancy rate of infertile couples^13^, but the etiology and pathogenesis of OA are still unclear, and improving the sperm quality of patients with OA remains challenging.

Traditional Chinese Medicine (TCM) has a long history of considering an individual or patient as an integral system with different statuses, focusing on multiple biological targets to produce therapeutic efficacies. TCMs are widely used to treat all kinds of diseases and conditions including OA^14^. Among them, *Morinda officinalis–Lycium barbarum* coupled-herbs (MOLBCH), which is composed of *Morinda officinalis* (MO) and *Lycium barbarum* (LB), has been widely used for treating OA over thousands of years. According to the concept of “ZHENG” and syndrome differentiation of the TCM theory^15,16^, MOLBCH is believed to possess the efficacies of tonifying the kidney and yang, storing essence, treating impotence and seminal emission. However, its unclear molecular mechanism greatly limits its clinical application. Therefore, it is of importance to reveal the bioactive components and potential targets of MOLBCH on OA.

As a novel approach to disentangle the different nature of diseases and the molecular mechanisms of medicine, network pharmacology is now drawing more and more attention in the field of TCM^17,18^. The concept of holism for TCM has much in common with the major points of network pharmacology, in which the general “one target, one drug” mode is shifted to a new “network target, multi-components” mode. In such a mode, the combination of network pharmacology and TCM would create a novel direction for discovering bioactive components and potential targets, revealing the molecular mechanism, and examining the scientific evidence of numerous herbs in TCM based on complex biological systems of human body. Molecular docking is a computational method in which small molecule ligands are docked to the active pockets of receptors (target proteins) to predict drug candidates. Correspondingly, integrating TCM, network pharmacology, and molecular docking can greatly accelerate the drug discovery and development as well.

Here, aiming at revealing the underlying molecular mechanisms of MOLBCH in the treatment of OA, we performed an integrated strategy based on network pharmacology and molecular docking to identify the bioactive components and potential targets. First, we obtained the MOLBCH and OA-related targets by searching various databases. Second, we constructed the MOLBCH component-target network and MOLBCH-OA common-target network to obtain the key bioactive components. Third, GO and KEGG pathway enrichment analyses of PPI network and clusters were performed to predict the core potential targets and important signaling pathways. Finally, the molecular docking was conducted to further verify the strong binding interactions between the key bioactive components and the core potential targets.

## Results

### MOLBCH component-target network

**Figure 1:**
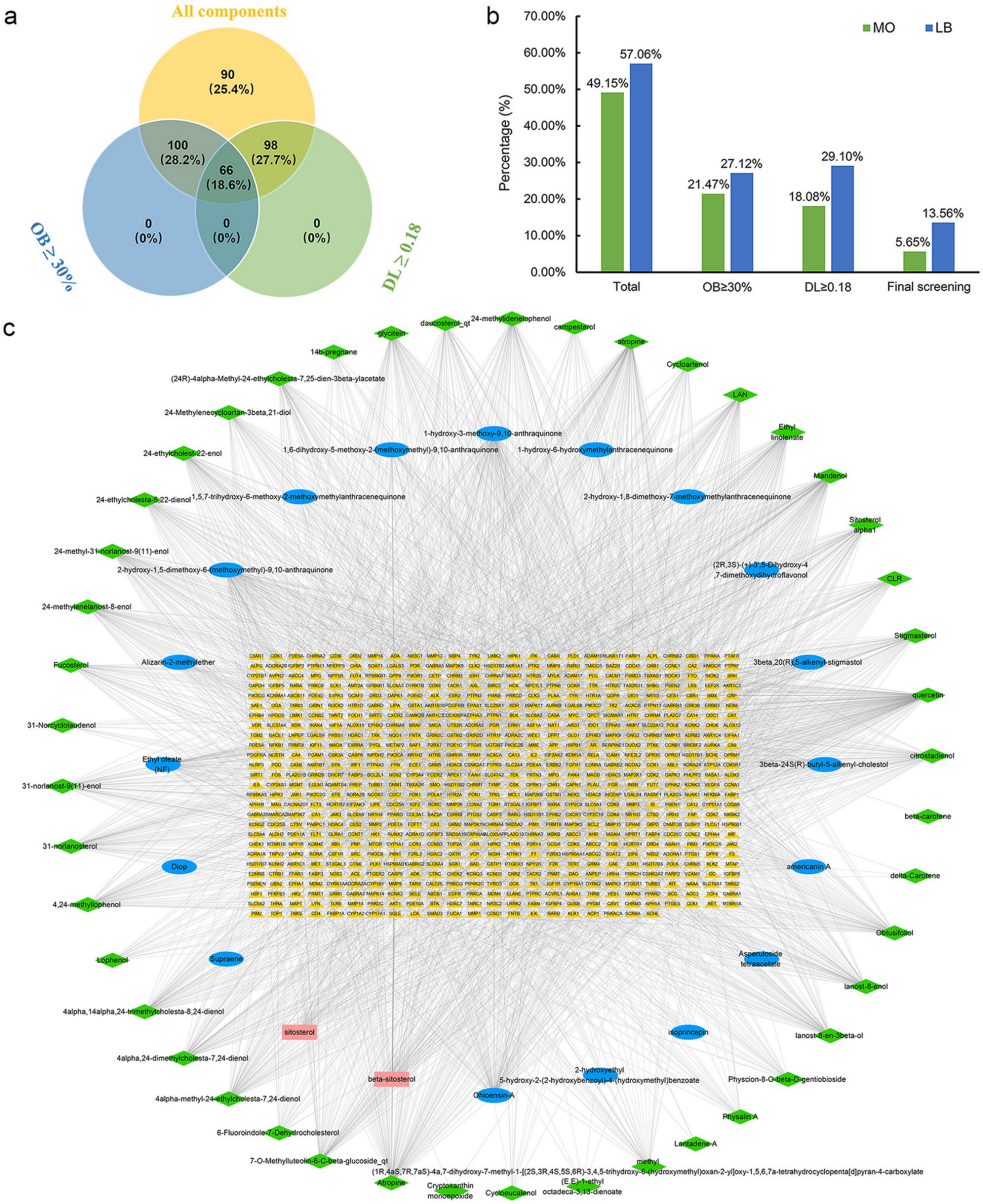
MOLBCH component-target network. (**a**) Venn diagram: 354 components (yellow section), and 66 bioactive components screened by two ADME-related models (blue section stands for the components of OB ≥ 30%, green section stands for DL ≥ 0.18). (**b**) Distributions of different herbs. (**c**) Construction of MOLBCH component-target visual network, including 736 nodes and 3034 edges. Green nodes and blue nodes stand for bioactive components from MO and LB respectively, yellow nodes stand for targets, pink nodes stand for beta-sitosterol and sitosterol. MO *Morinda officinalis*, LB *Lycium barbarum*.

354 components of MOLBCH were obtained from TCMSP and TCMID. Among them, 174 components were from MO, 202 components were from LB, and 22 common components were from MO and LB. Subsequently, OB ≥ 30% and DL ≥ 0.18 were used as the screening criteria. Finally, we got 66 bioactive components of MOLBCH, including 20 from MO, 48 from LB, and 2 (beta-sitosterol, sitosterol) from MO and LB (Fig. 1a,b). Then, the structural information of 66 bioactive components was collected from PubChem and ALOGPS2.1 (Supplementary Table S2). Four public webservers, Swiss Target Prediction, SEA, TCMSP and Drugbank, were used to predict the potential targets of the bioactive components according to the similarity-based method. 671 potential targets were predicted from 65 obtained bioactive components after removing duplicates, except one non-effective component (cyanin). The MOLBCH component-target network was constructed by Cytoscape software, including 736 nodes and 3034 edges (Fig. 1c). Among the obtained nodes, 372 targets were from 20 components of MO, 504 targets were from 47 components of LB, and 205 common targets were from the common components of MO and LB. According to these results, we suggest that MO and LB act on an integrated effect on OAthrough the common targets to a certain extent.

### MOLBCH-OA common-target network

**Figure 2:**
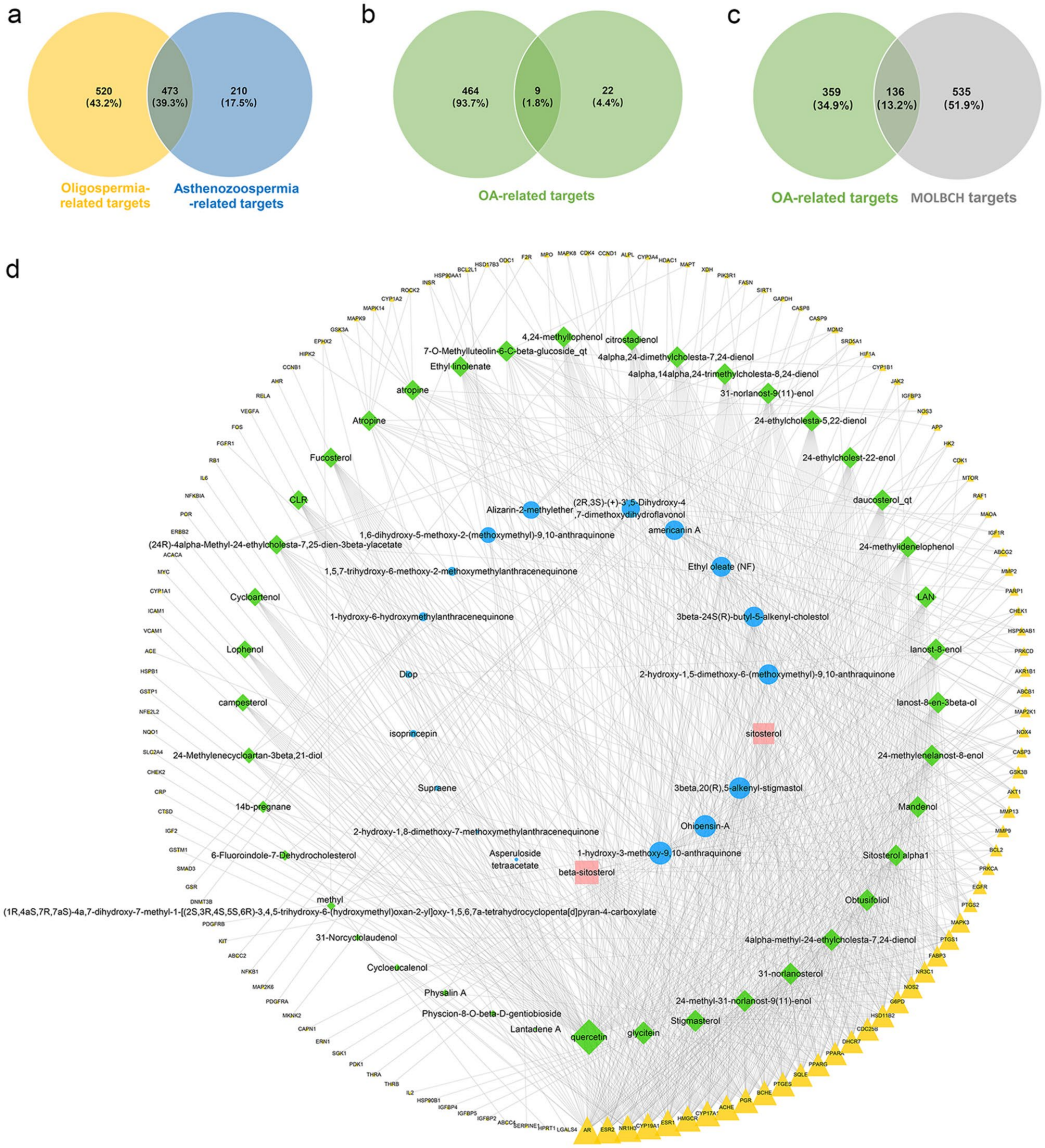
MOLBCH-OA common-target network. (**a**) Intersection of Venn diagram: 473 targets are common to oligospermia and asthenozoospermia. (**b**) Union of Venn diagram: OA-related targets are 495, including 473 common targets between oligospermia and asthenozoospermia, and 31 targets from the search term “oligoasthenozoospermia”. (**c**) Intersection of Venn diagram: 136 targets are common to OA and MOLBCH. (**d**) Common-target network, including 196 nodes and 900 edges. The size of the circle represents the node degree of the target protein. Green nodes and blue nodes stand for bioactive components from MO and LB respectively, yellow nodes stand for targets, pink nodes stand for beta-sitosterol and sitosterol. OA, oligoasthenozoospermia; MOLBCH, *Morinda officinalis*-*Lycium barbarum* coupled-herbs.

The pathogenesis of OA is resulted from the co-occurring condition of oligospermia and asthenozoospermia. Therefore, taking the genes of these two diseases into consideration is essential to reveal the common targets of MOLBCH on OA. The number of targets got from oligospermia and asthenozoospermia is 993 and 683, while 473 overlapping targets were collected (Fig. 2a). 31 targets from the search term “oligoasthenozoospermia” were added to get total 495 OA-related targets (Fig. 2b). In order to ensure the comprehensiveness of target collection, we used five human genomic databases, namely DisGeNET, CTD, OMIM, GeneCards and NCBI. The number of targets from these databases was 51, 415, 36, 78 and 30, respectively.

Additionally, 136 common targets were obtained from the intersection of OA-related targets and MOLBCH targets (Fig. 2c). 19 bioactive components from MO and 43 bioactive components from LB were associated with 136 MOLBCH-OA common targets (Fig. 2d). Furthermore, the degree value of the MOLBCH-OA common-target network was calculated by Network Analyzer, a plugin of Cytoscape, which was used as a screening condition to get the key bioactive components (Supplementary Table S6). We found four key bioactive components of the MOLBCH-OA common-target network, namely Ohioensin-A from MO, quercetin from LB, beta-sitosterol and sitosterol from both MO and LB, respectively. Meanwhile, the degree value of Androgen receptor (AR), Estrogen receptor beta (ESR2), Nuclear receptor subfamily 1 group H member 3 (NR1H3), Cytochrome P450 19A1 (CYP19A1), Estrogen receptor (ESR1) was high, indicating that these five targets played an important role in MOLBCH-OA common-target network.

### MOLBCH-OA PPI network and evaluation

**Figure 3:**
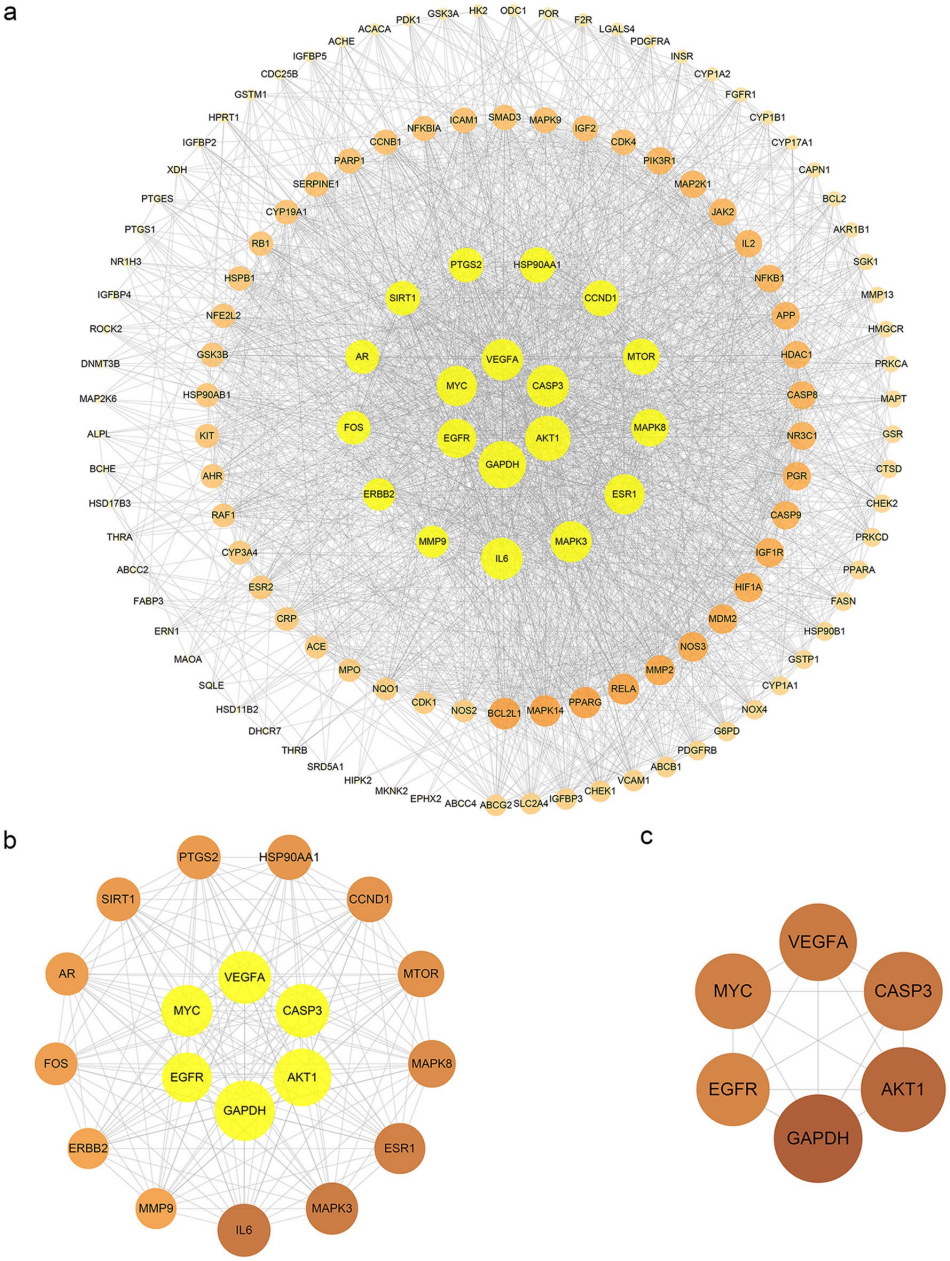
MOLBCH-OA PPI Network and Evaluation. (**a**) MOLBCH-OA PPI network (136 nodes and 2233 edges). (**b**) PPI network by the screening criteria of ‘DC ≥ 58′ (19 nodes and 169 edges). (**c**) Core-target PPI network by the screening criteria of “‘DC’ ≥ 75, ‘EC’ ≥ 0.1584286, ‘LAC’ ≥ 33.873016, ‘BC’ ≥ 400.05, ‘CC’ ≥ 0.6818182, ‘NC’ ≥ 62.32288, ‘SC’ ≥ 12,901,748,000,000,000,000, ‘IC’ ≥ 15.5665245” (6 nodes and 15 edges). The nodes stand for the target protein of MOLBCH on OA. The color scales and the size of the circle represent the node degree of the target protein.

To elucidate the protein interactions of the 136 MOLBCH-OA common targets, the STRING database was employed here. The confidence score of the protein–protein interaction (PPI) information was set to 0.4 or higher, and the Cytoscape software was used to visualize the PPI network (Fig. 3a). The Degree (DC) of the PPI network were calculated by Network Analyzer. The topological parameters (Betweenness (BC), Closeness (CC), Eigenvector (EC), Local Average Connectivity-based method (LAC), Network (NC), Subgragh (SC), Information (IC)) of the PPI network were calculated by CytoNCA (Fig. 3b,c) (Supplementary Table S7). The results showed that Glyceraldehyde-3-phosphate dehydrogenase (GAPDH), RAC-alpha serine/threonine-protein kinase (AKT1), Caspase-3 (CASP3), Vascular endothelial growth factor A (VEGFA), Myc proto-oncogene protein (MYC), Epidermal growth factor receptor (EGFR) were the most vital targets of MOLBCH-OA PPI Network (Fig. 3c).

### GO and KEGG pathway enrichment analyses of MOLBCH-OA PPI network

**Figure 4:**
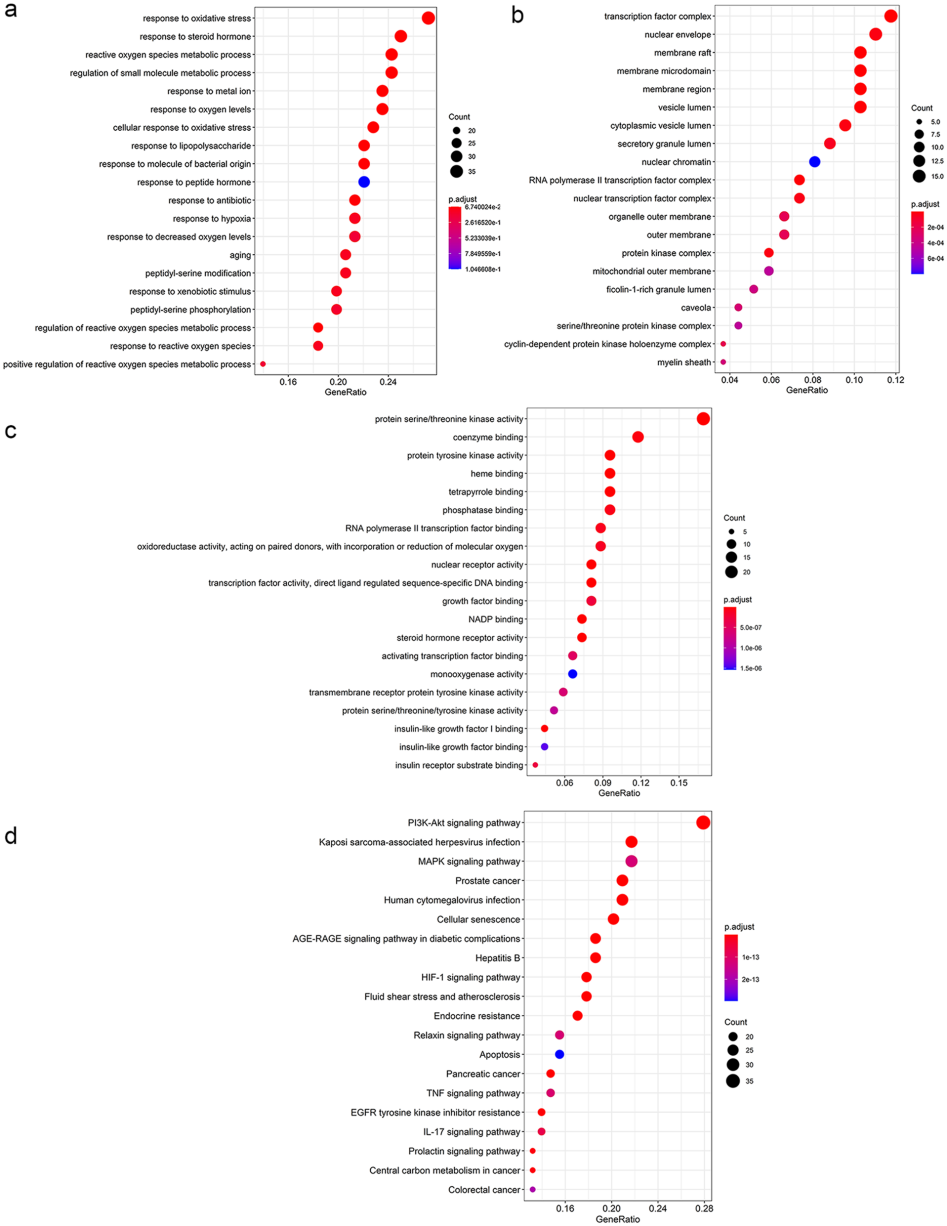
GO and KEGG pathway enrichment analyses of MOLBCH-OA PPI Network (p-value ≤ 0.05). (**a**) The top 20 biological processes. (**b**) The top 20 cellular components. (**c**) The top 20 molecular functions. (**d**) The top 20 KEGG pathways. The color scales indicate the different thresholds for the p-values, and the sizes of the dots represent the number of genes corresponding to each term.

The significant functions derived from MOLBCH-OA PPI network was explored by the GO and KEGG pathway enrichment analyses. Firstly, GO enrichment analysis was conducted, including biological process (BP), cellular component (CC), and molecular function (MF) (Supplementary Table S9). The top 20 significant terms are shown in Fig. 4a–d. Specifically, BP is related to “response to oxidative stress (OS)”, “reactive oxygen species metabolic process”, “response to oxygen levels”, “cellular response to oxidative stress”, “response to hypoxia”, “response to decreased oxygen levels”, “regulation of reactive oxygen species metabolic process”, “response to reactive oxygen species” and “positive regulation of reactive oxygen species metabolic process”, indicating that MOLBCH has an anti-oxidant effect to regulate male reproduction on OA (Fig. 4a). CC is associated with “nuclear envelope”, “membrane raft”, “membrane microdomain”, “membrane region”, “organelle outer membrane”, “outer membrane”, “mitochondrial outer membrane”, “transcription factor complex”, “nuclear chromatin”, “RNA polymerase II transcription factor complex”, “protein kinase complex”, “serine/threonine protein kinase complex” and “cyclin-dependent protein kinase holoenzyme complex”, demonstrating that MOLBCH can regulate male reproductive function by acting on membrane and protein kinase complex in cellular (Fig. 4b). MF is relevant to “protein serine/threonine kinase activity”, “protein tyrosine kinase activity”, “transmembrane receptor protein tyrosine kinase activity” and “protein serine/threonine/tyrosine kinase activity”, revealing that MOLBCH could affect protein kinase activity in the pathogenesis of OA (Fig. 4c).

The KEGG database was conducted to investigate the pathways related to the possible functions of the PPI network (Supplementary Table S9). The top 20 significant pathways were shown in Fig. 4d. The results indicate that MOLBCH regulates apoptosis process through “PI3K-Akt signaling pathway”, “MAPK signaling pathway”, “Apoptosis”, “IL-17 signaling pathway” “TNF signaling pathway”. In addition, “AGE-RAGE signaling pathway in diabetic complications” and “HIF-1 signaling pathway” are related to oxidant stress in the course of disease. Besides, MOLBCH could regulate the male reproductive function by affecting “Prostate cancer”, “Endocrine resistance”, “Relaxin signaling pathway”, “EGFR tyrosine kinase inhibitor resistance” and “Prolactin signaling pathway”. Therefore, through the GO and KEGG pathway enrichment analyses of MOLBCH-OA PPI network, we believe that MOLBCH might treat OA via promoting male productive function, reducing OS, and inhibiting apoptosis process.

### GO and KEGG pathway enrichment analyses of the cluster

**Figure 5:**
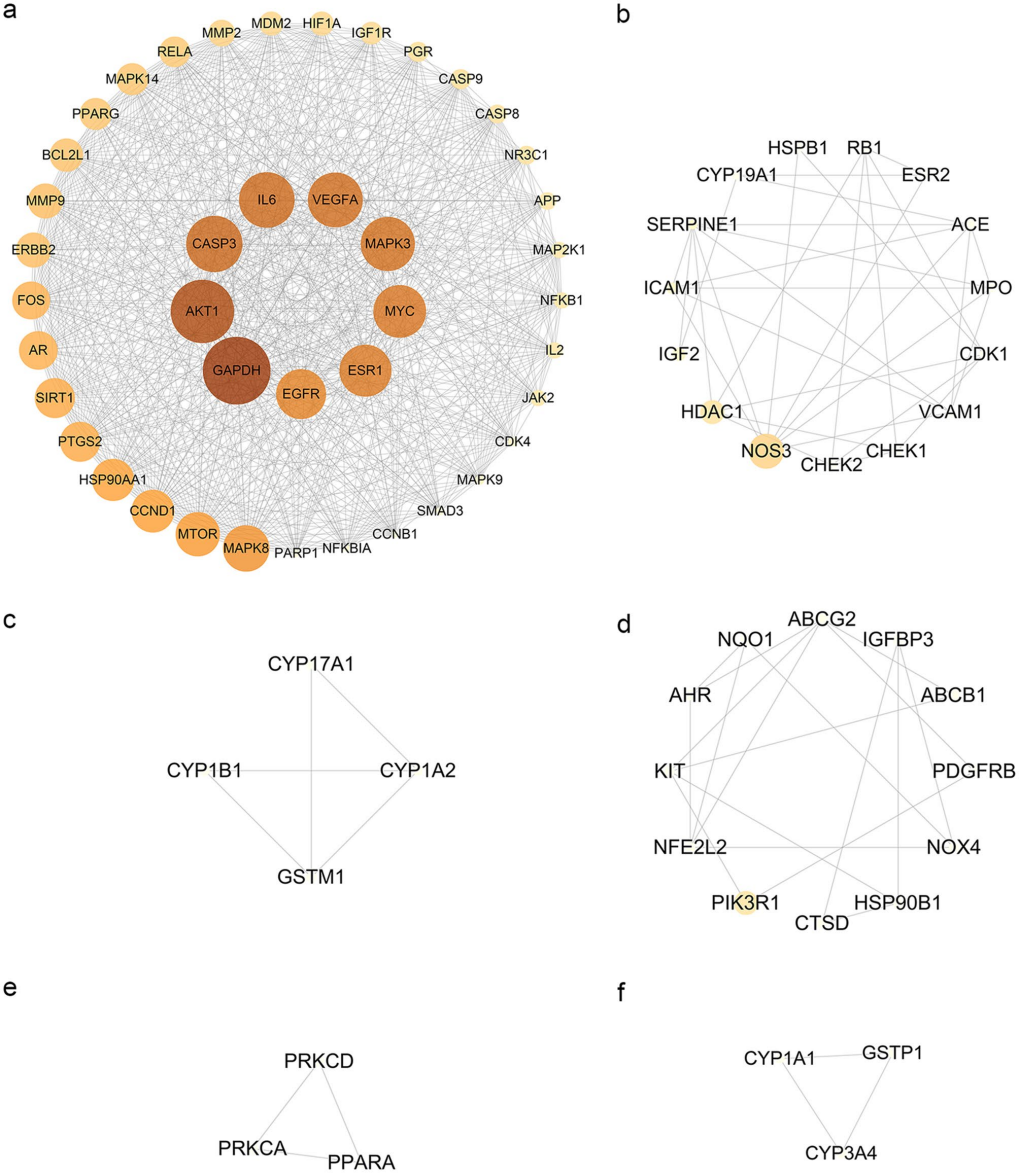
Cluster analysis of MOLBCH-OA PPI Network. (**a**) Cluster 1, composed of 42 nodes and 724 edges (score = 35.317). (**b**) Cluster 2, composed of 15 nodes and 34 edges (score = 4.857). (**c**) Cluster 3, composed of 4 nodes and 5 edges (score = 3.333). (**d**) Cluster 4, composed of 12 nodes and 18 edges (score = 3.273). (**e**) Cluster 5, composed of 3 nodes and 3 edges (score = 3). (**f**) Cluster 6, composed of 3 nodes and 3 edges (score = 3). The nodes stand for the target protein of MOLBCH on OA. The color scales and the size of the circle represent the node degree of the target protein.

Network cluster is defined as a set of highly interconnected nodes, which is helpful to discover and reveal the hidden biological information in the network^19^. In order to identify the potential mechanism of the 136 common targets, the MOLBCH-OA PPI network was divided into 6 clusters (Fig. 5). According the result of Network Analyzer, the degree value of GAPDH, AKT1, CASP3, Interleukin-6 (IL6), VEGFA, Mitogen-activated protein kinase 3 (MAPK3), Myc proto-oncogene protein (MYC), ESR1, and Epidermal growth factor receptor (EGFR) is much higher than other proteins in cluster 1.

**Figure 6:**
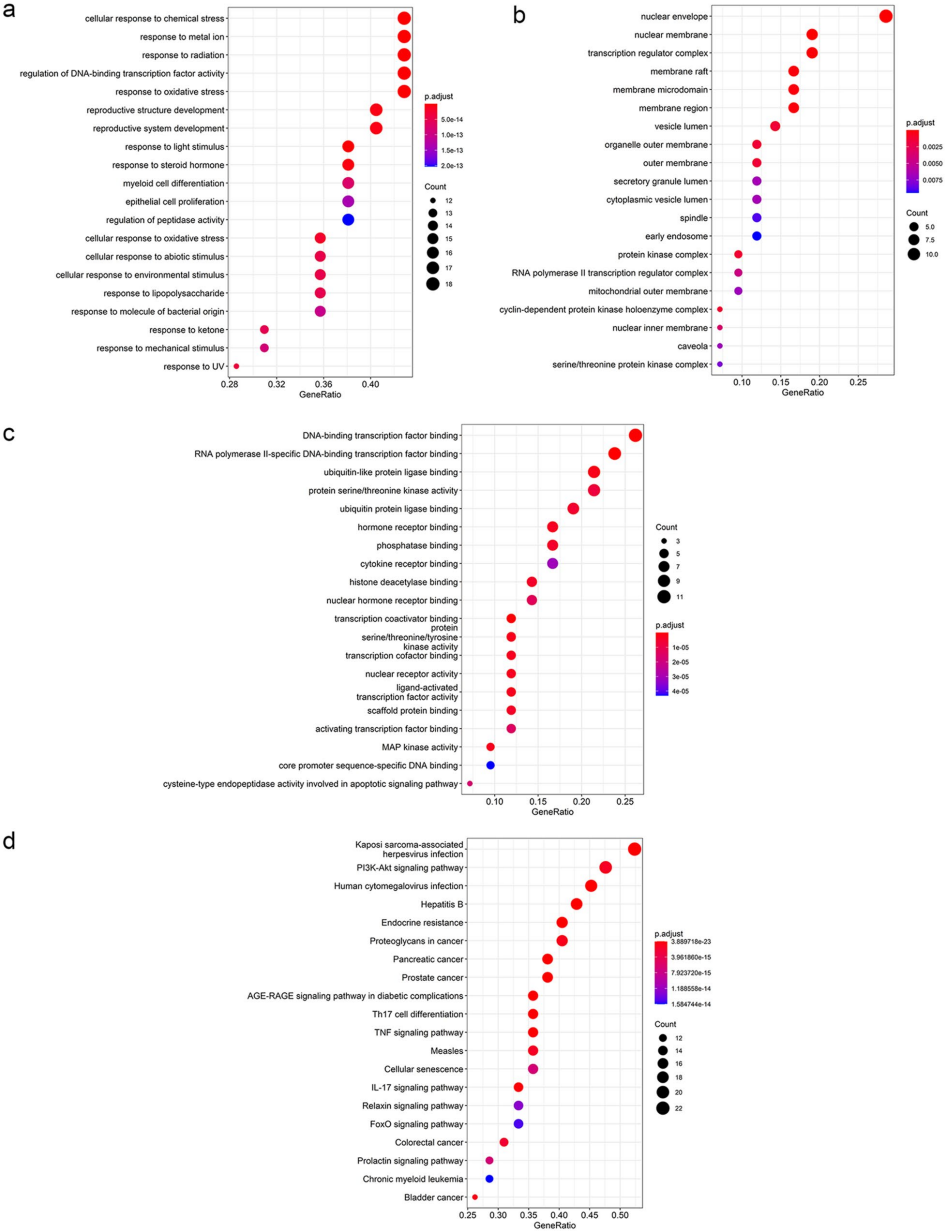
GO and KEGG pathway enrichment analyses of cluster 1 (p-value ≤ 0.05). (**a**) The top 20 biological processes for cluster 1. (**b**) The top 20 cellular components for cluster 1. (**c**) The top 20 molecular functions for cluster 1. (**d**) The top 20 KEGG pathways for cluster 1. The color scales indicate the different thresholds for the p-values, and the sizes of the dots represent the number of genes corresponding to each term.

Since the score of cluster 1 was much higher than other clusters, so we performed GO and KEGG pathway enrichment analyses to further investigate the proteins of cluster 1 (Fig. 6). “Response to OS”, “cellular response to OS”, “reproductive structure development”, “reproductive system development” in BP suggest that the proteins in cluster 1 are related to anti-oxidant effect and male reproductive regulation (Fig. 6a). “Nuclear envelope”, “nuclear membrane”, “membrane raft”, “membrane microdomain”, “membrane region”, “organelle outer membrane”, “outer membrane”, “mitochondrial outer membrane”, “nuclear inner membrane”, “protein kinase complex”, “RNA polymerase II transcription factor complex” and “cyclin-dependent protein kinase holoenzyme complex” in CC demonstrate that the proteins in cluster 1 are relevant to membrane and protein kinase complex in cellular (Fig. 6b). “DNA-binding transcription factor binding”, “RNA polymerase II-specific DNA-binding transcription factor binding”, “activating transcription factor binding” and “core promoter sequence-specific DNA binding” in MF reveals that the proteins in cluster 1 could affect DNA binding (Fig. 6c).

The KEGG database was conducted to investigate the pathways related to the possible functions of the PPI network (Supplementary Table S10). The top 20 significant KEGG pathways were shown in Fig. 6d. The KEGG results indicate that the proteins in cluster 1 are related to inhibit apoptosis process through “PI3K-Akt signaling pathway” and “IL-17 signaling pathway”. The proteins in cluster 1 also have connections with promoting male reproductive function by affecting “Prostate cancer”, “Endocrine resistance”, “Relaxin signaling pathway” and “Prolactin signaling pathway”. The GO and KEGG pathway enrichment analyses indicate that the proteins in cluster 1 are mainly related to oxidant stress, apoptosis, and male reproductive function, which consistents with the results of MOLBCH-OA PPI network analysis (Fig. 6d–g).

### Core component-target-pathway network

**Figure 7:**
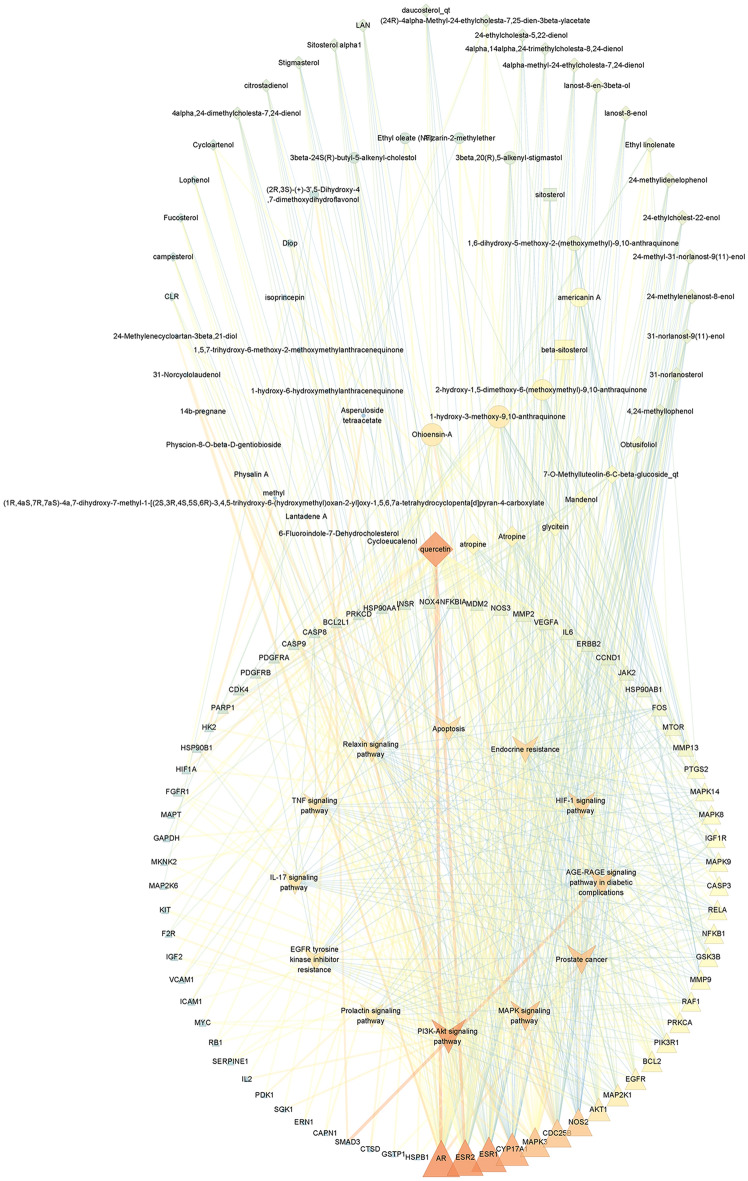
Core component-target-pathway network. The ellipse nodes represent ingredients from MO, and the diamond nodes represent ingredients from LB. The rectangle nodes represent beta-sitosterol and sitosterol. The triangle nodes represent the core targets from MOLBCH and OA. The V nodes represent 12 signaling pathways from enrichment analysis. The size of the circle represents the node degree of the target protein. The color scales from orange to blue indicate the value from high to low of the node degree and the edge betweeness.

In order to gain a holistic understanding of the underlying mechanism of OA, core component-target-pathway network was constructed by Cytoscape software. As shown in Fig. 7, a total of 143 nodes and 646 edges were calculated, and 17 MO-related components and 43 LB-related components were identified. According to the reported literature^20–23^ and the degree value obtained from Network Analyzer, the most remarkable components corresponding to MO and LB are Ohioensin-A and quercetin, respectively. The common ingredients beta-sitosterol and sitosterol from MO and LB is also of great importance during the course of drug treatment.

**Figure 8:**
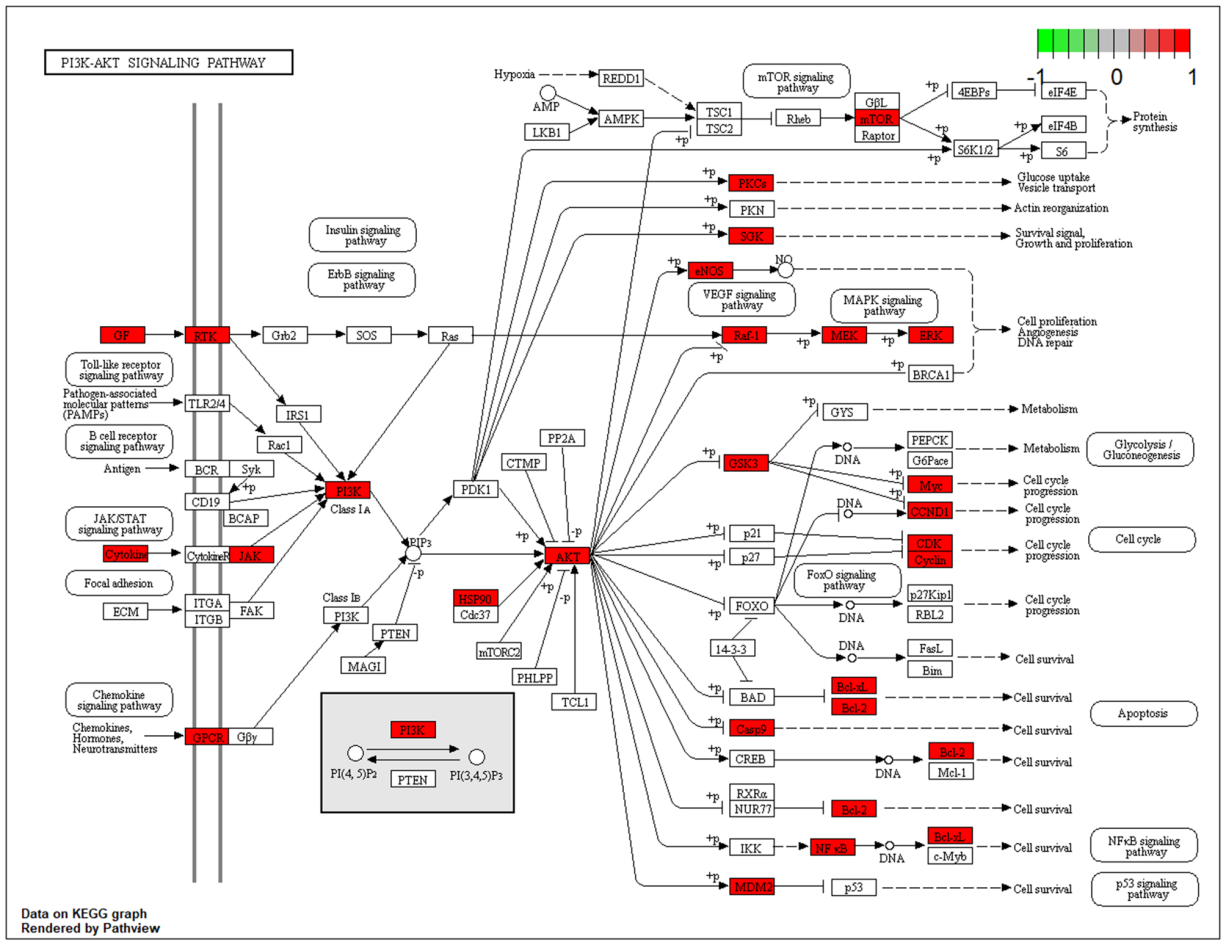
PI3K-AKT signaling pathway. The red rectangle represents the targets related to the core component-target-pathway network.

**Figure 9:**
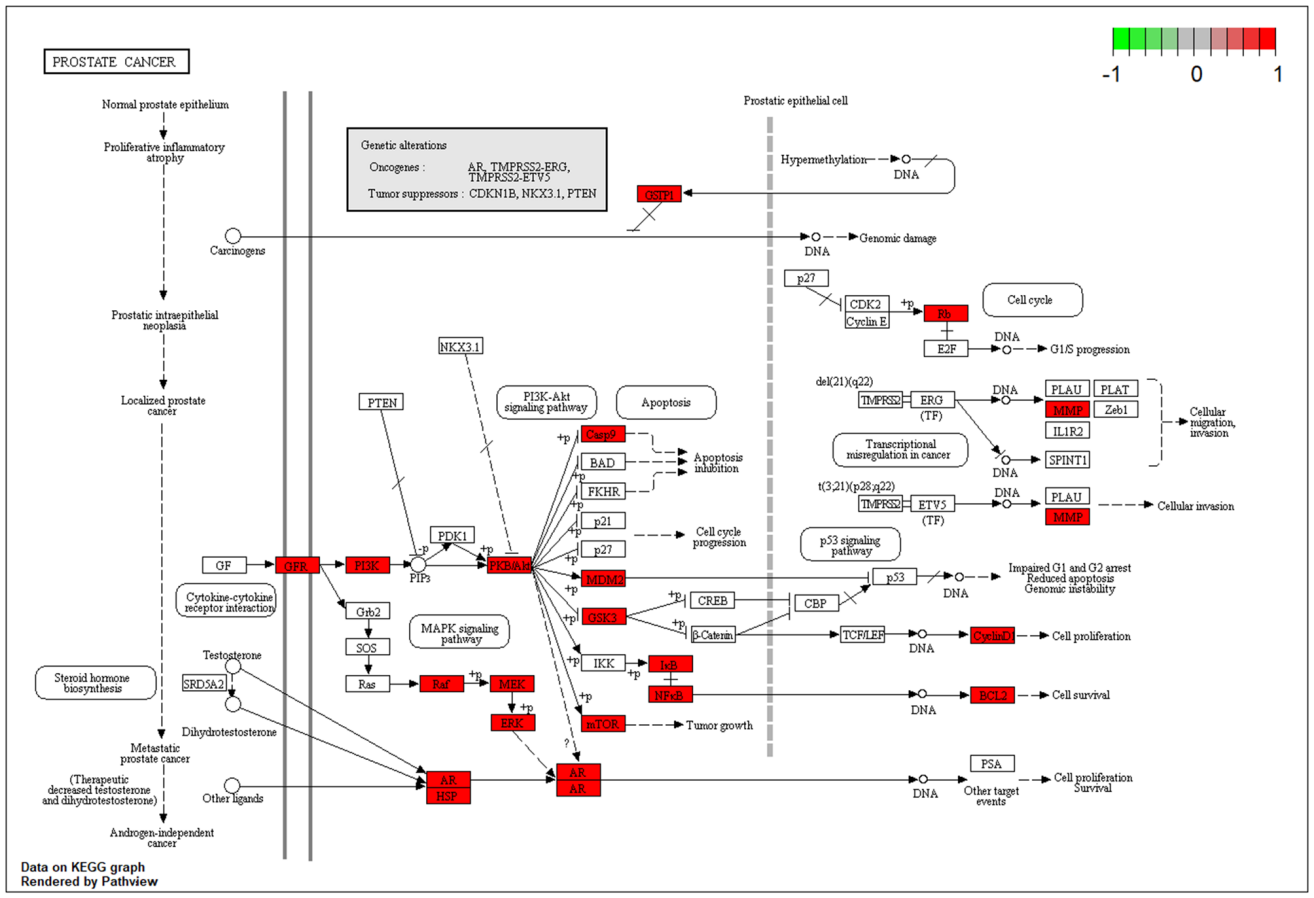
Prostate cancer. The red rectangle represents the targets related to the core component-target-pathway network.

**Figure 10:**
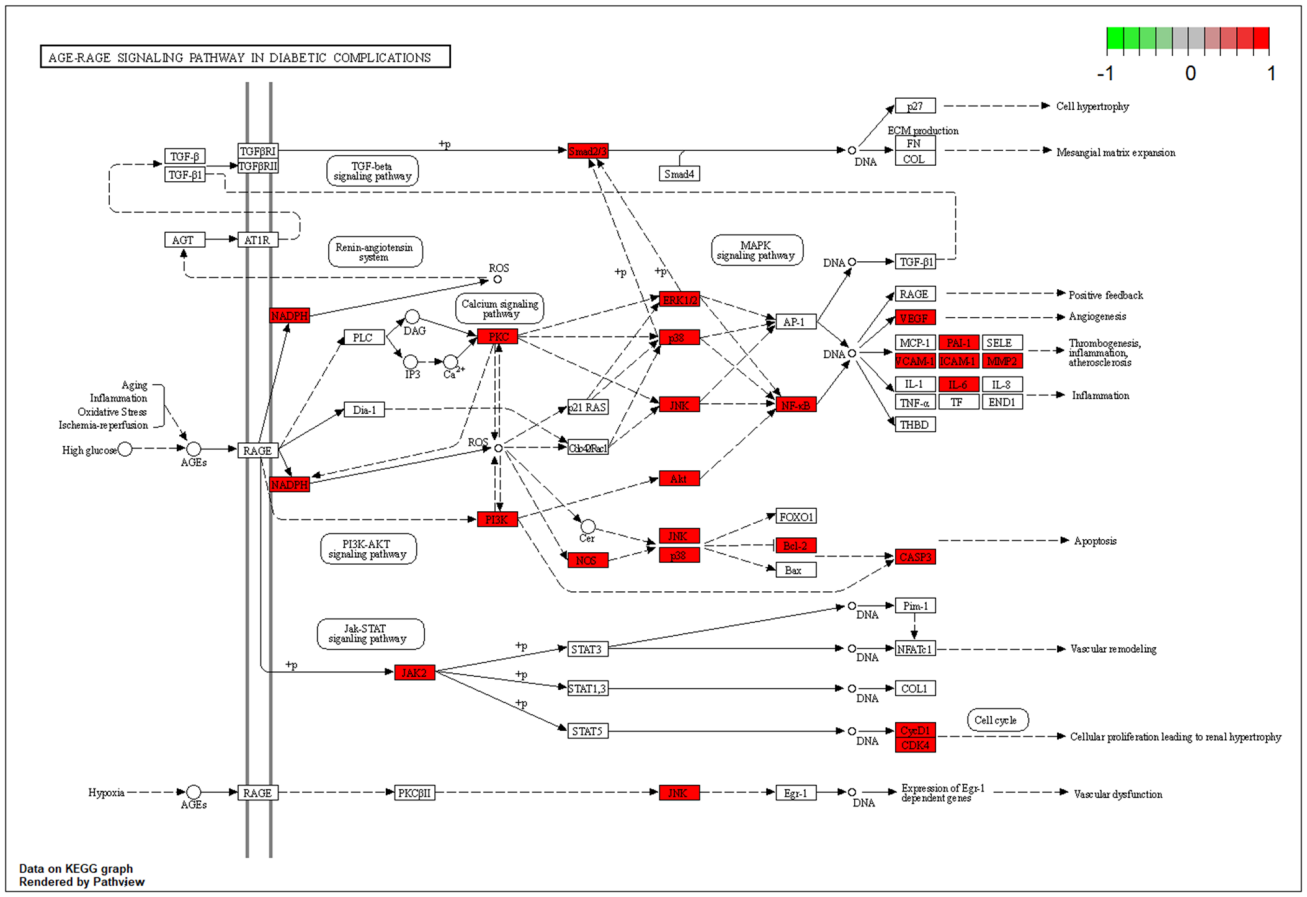
AGE-RAGE signaling pathway in diabetic complications. The red rectangle represents the targets related to the core component-target-pathway network.

As shown in Table 1, GO and KEGG analyses of MOLBCH-OA PPI network and clusters indicate that MOLBCH could treat OA by regulating male reproductive function, reducing apoptosis and OS. The main KEGG signaling pathways related to the above mechanism are PI3K-Akt signaling pathway, MAPK signaling pathway, Apoptosis, IL-17 signaling pathway, TNF signaling pathway, AGE-RAGE signaling pathway in diabetic complications, HIF-1 signaling pathway, Prostate cancer, Endocrine resistance, Relaxin signaling pathway, EGFR tyrosine kinase inhibitor resistance. The targets relevant to the above pathways were shown in Fig. 6, AR, ESR2, ESR1, Cytochrome P450 17A1 (CYP17A1), MAPK3, M-phase inducer phosphatase 2 (CDC25B), Nitric oxide synthase (NOS2), AKT1, Dual specificity mitogen-activated protein kinase kinase 1 (MAP2K1), and EGFR are the top 10 vital targets in core-component-target-pathway network. In addition, GAPDH, AKT1, CASP3, VEGFA, MYC, EGFR, IL6, MAPK3, and ESR1 also play an essential role in MOLBCH-OA PPI network and clusters. We found that the duplicated targets between them are ESR1, MAPK3, AKT1, and EGFR. Since AR is the most significant target in MOLBCH-OA common-target network and core component-target-pathway network, GAPDH is the most significant in the MOLBCH-OA PPI network and clusters, we suggest that AR, ESR1, MAPK3, AKT1, and GAPDH are the core potential targets of MOLBCH against OA. Based on the above results, we hold that PI3K-Akt signaling pathway, Prostate cancer, and AGE-RAGE signaling pathway in diabetic complications are the most important signaling pathways during the process of MOLBCH in treating OA. The targets related to these three signaling pathways are shown in Figs. 8, 9 and 10. Furthermore, we chose Ohioensin-A, quercetin, beta-sitosterol, sitosterol as the molecular docking ligands, AR, ESR1, MAPK3, AKT1, and GAPDH as the targets to reveal the interaction between the key bioactive components and core potential targets of MOLBCH against OA.

**Table 1:** Main KEGG signaling pathways and represent core targets.

Classification	KEGG signaling pathways	Core targets
Apoptosis	PI3K-Akt signaling pathway	AKT1, MAPK3
	MAPK signaling pathway	
	Apoptosis	
	IL-17 signaling pathway	
	TNF signaling pathway	
Male reproductive function	Prostate cancer	AR, ESR1
	Endocrine resistance	
	Relaxin signaling pathway	
	EGFR tyrosine kinase inhibitor resistance	
	Prolactin signaling pathway	
OS	AGE-RAGE signaling pathway in diabetic complications	GAPDH
	HIF-1 signaling pathway

### Molecular docking

Molecular docking is a procedure using molecular modeling techniques to predict how a protein interacts with small molecules (ligands)^24^. In this study, we chose Ohioensin-A from MO, quercetin from LB, and the common ingredients beta-sitosterol and sitosterol from MO and LB as small molecules (ligands), AR, ESR1, MAPK3, AKT1, and GAPDH as proteins to perform the molecular docking. These proteins not only play an important role in the KEGG signaling pathways, but also serve as the key nodes of the PPI network and clusters. The obtained docking results indicate that the receptor-ligand interaction between drugs and proteins includes hydrophobic interactions and polar interactions. According to Tables 2 and 3, Ohioensin-A, quercetin, beta-sitosterol and sitosterol have strong binding interactions with AR, ESR1, MAPK3, AKT1, and GAPDH.

**Table 2:** Results of molecular docking between the bioactive components and the core predicted targets.

Ligand	Proteins	Residues	Hydrogen bonds
Ohioensin-A	AR	Leu880, Asn705, Met780, Phe876, Leu704, Thr877, Leu701, Leu873, Val746, Trp741, Phe764, Gly708, Met745, Met749, Gln711, Met787	Met895 (3.1 Å)
	ESR1	Arg394, Leu391, Phe404, Leu384, Leu387, Glu353, Leu428, Ile424, Leu346, Ala350, Met421, Met343, Thr347, Leu540, Leu525	His524 (3.0 Å)
	MAPK3	Ser286, Pro285, Lys287, Gly262, Leu284, Asn255, Leu258	Ser263 (2.7 Å)
	AKT1	His194, Glu198, Gly294, Thr195, Lys179, Lys163, Leu181, Gly162, Val164, Gly159 and Phe161	Thr160 (3.1 Å)
	GAPDH	Ala183, Arg13, Asn316, Ile14, Cys152, Ala123	Gly100 (2.9 Å)
quercetin	AR	Thr877, Met780, Phe876, Leu701, Leu880, Asn705, Phe764, Leu704, Met 749, Met787, Gly708, Val746, Gln711, Leu707	Leu873 (2.7 Å), Met745 (3.1 Å)
	ESR1	Leu391, Phe404, Ala350, Ile424, Met421, His524, Leu525, Leu384, Leu349, Leu346 and Glu353	Leu387 (2.8 Å), Arg394 (3.3 Å), Arg394 (2.7 Å), Gly521 (2.5 Å)
	MAPK3	Lys287, Leu258, Leu284, Asn255, Gly259, Gly262	Pro285 (3.0 Å), Ser263 (2.8 Å)
	AKT1	Glu278, Lys276, Phe161, Glu191, His194, Gly294, Leu295, Asp292	Asp274 (2.9 Å), Ser7 (2.9 Å), Thr5 (3.0 Å)
	GAPDH	Pro36, Phe37, Thr99, Gly12, Ser98, Gly10, Asn9	Asp35 (2.8 Å), Asn34 (3.0 Å)
beta-sitosterol	AR	Phe891, Leu880, Leu701, Met780, Thr877, Trp741, Leu873, Met745, Met742, Met787, Met749, Arg752, Gln711, Val746, Gly708, Leu707, Leu704, Phe764, Met895	Asn705 (3.0 Å)
	ESR1	Arg394, Leu387, Leu391, Leu349, Leu428, Ile424, Met388, Met421, His524, Gly521, Leu384, Leu346, Leu525, Thr347, Met343, Ala350	Phe404 (3.1 Å), Glu353 (2.4 Å)
	MAPK3	Ser283, Pro285, Gly259, Leu258, Asn255, Leu284 and Tyr280	–
	AKT1	Asn279, Gly294, Leu295, Glu191, Phe161, His194, Leu181, Glu198, Lys179, Thr195, Asp292, Val164	Glu234 (3.0 Å)
	GAPDH	Phe37, Ile38, Ala183, Ile14, Glu317, Asn316, Thr182, Cys152, Tyr320, Arg13, Thr99, Gly12 and Ser98	Asp35 (3.1 Å)
sitosterol	AR	Thr877, Met780, Met895, Phe891, Leu880, Met742, Leu701, Leu873, Trp741, Val746, Met745, Met787, Met749, Leu707, Arg752, Gly708, Gln711, Phe764, Leu704	Asn705 (2.9 Å)
	ESR1	Leu387, Leu391, Leu428, Met388, Ile424, His524, Gly521, Leu384, Met421, Leu525, Trp383, Met343, Thr347, Leu346, Glu353, Ala350, Leu349	Arg394 (2.1 Å), Phe404 (3.1 Å)
	MAPK3	Tyr280, Asn255, Lys287, Pro285, Gly259, Leu258, Leu284, Ser283	–
	AKT1	Val164, Asn279, Glu191, Gly294, Leu295, Leu181, Glu198, His194, Phe161, Thr195, Lys179, Asp292	Glu234 (3.0 Å)
	GAPDH	Ser122, Ile14, Arg13, Ala183, Gly12, Asp35, Phe37, Ser98, Thr99	Cys152 (3.2 Å)

**Table 3:** The binding energy of molecular docking between the bioactive components and the core predicted targets.

Ligand	Proteins	Affinity	Dist from best mode	
		(kcal/mol)	rmsd l.b	rmsd u.b
Ohioensin-A	AR	4.5	0.000	0.000
	ESR1	− 4.7	0.000	0.000
	MAPK3	− 6.2	0.000	0.000
	AKT1	− 9.9	0.000	0.000
	GAPDH	− 8.3	0.000	0.000
Quercetin	AR	− 8.0	0.000	0.000
	ESR1	− 6.9	0.000	0.000
	MAPK3	− 5.3	0.000	0.000
	AKT1	− 7.7	0.000	0.000
	GAPDH	− 6.5	0.000	0.000
Beta-sitosterol	AR	− 0.4	0.000	0.000
	ESR1	− 4.5	0.000	0.000
	MAPK3	− 5.4	0.000	0.000
	AKT1	− 9.8	0.000	0.000
	GAPDH	− 7.8	0.000	0.000
Sitosterol	AR	− 1.2	0.000	0.000
	ESR1	− 4.6	0.000	0.000
	MAPK3	− 5.7	0.000	0.000
	AKT1	− 9.6	0.000	0.000
	GAPDH	− 7.2	0.000	0.000

**Figure 11:**
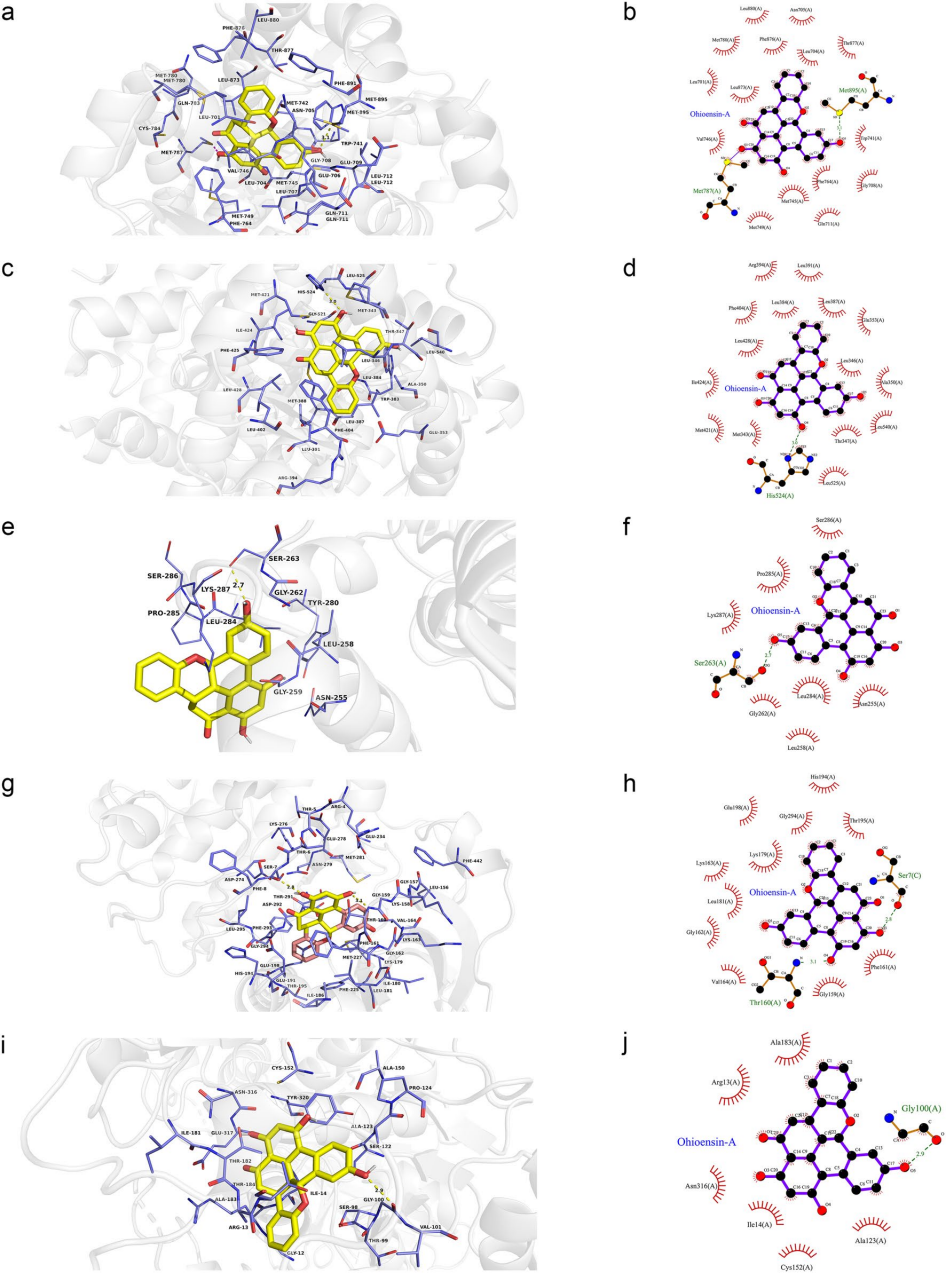
Molecular models of the binding of Ohioensin-A from MO to the predicted targets (**a**, **b**) AR, (**c**, **d**) ESR1, (**e**, **f**) MAPK3, (**g**, **h**) AKT1 and (**i**, **j**) GAPDH shown as 3D diagrams and 2D diagrams.

Ohioensin-A was docked with sixteen residues to form hydrophobic interactions in AR (Leu880, Asn705, Met780, Phe876, Leu704, Thr877, Leu701, Leu873, Val746, Trp741, Phe764, Gly708, Met745, Met749, Gln711 and Met787) and hydrogen bond (Ohioensin-A_O5_: Met895_SD_ (3.1 Å)) (Fig. 11a,b). In addition, Ohioensin-A was predicted to interact with ESR1 via Arg394, Leu391, Phe404, Leu384, Leu387, Glu353, Leu428, Ile424, Leu346, Ala350, Met421, Met343, Thr347, Leu540, Leu525, and form hydrogen bond with the residue His524 (3.0 Å) (Fig. 11c,d). Ohioensin-A could bind to MAPK3 by forming hydrophobic interactions with the neighboring residues Ser286, Pro285, Lys287, Gly262, Leu284, Asn255, Leu258 and hydrogen bond with Ser263 (2.7 Å) (Fig. 11e,f). Besides, Ohioensin-A bound to a pocket in AKT1, composing of His194, Glu198, Gly294, Thr195, Lys179, Lys163, Leu181, Gly162, Val164, Gly159 and Phe161. The hydrogen bond formed by Ohioensin-A_O4_ andThr160_N_ (3.1 Å), further enhances the interaction between the ligand and the AKT1 protein (Fig. 11g,h). Furthermore, Ohioensin-A was docked to GAPDH by forming hydrophobic interactions with the neighboring residues (Ala183, Arg13, Asn316, Ile14, Cys152, Ala123) and hydrogen bond with Gly100 (2.9 Å) (Fig. 11i,j).

**Figure 12:**
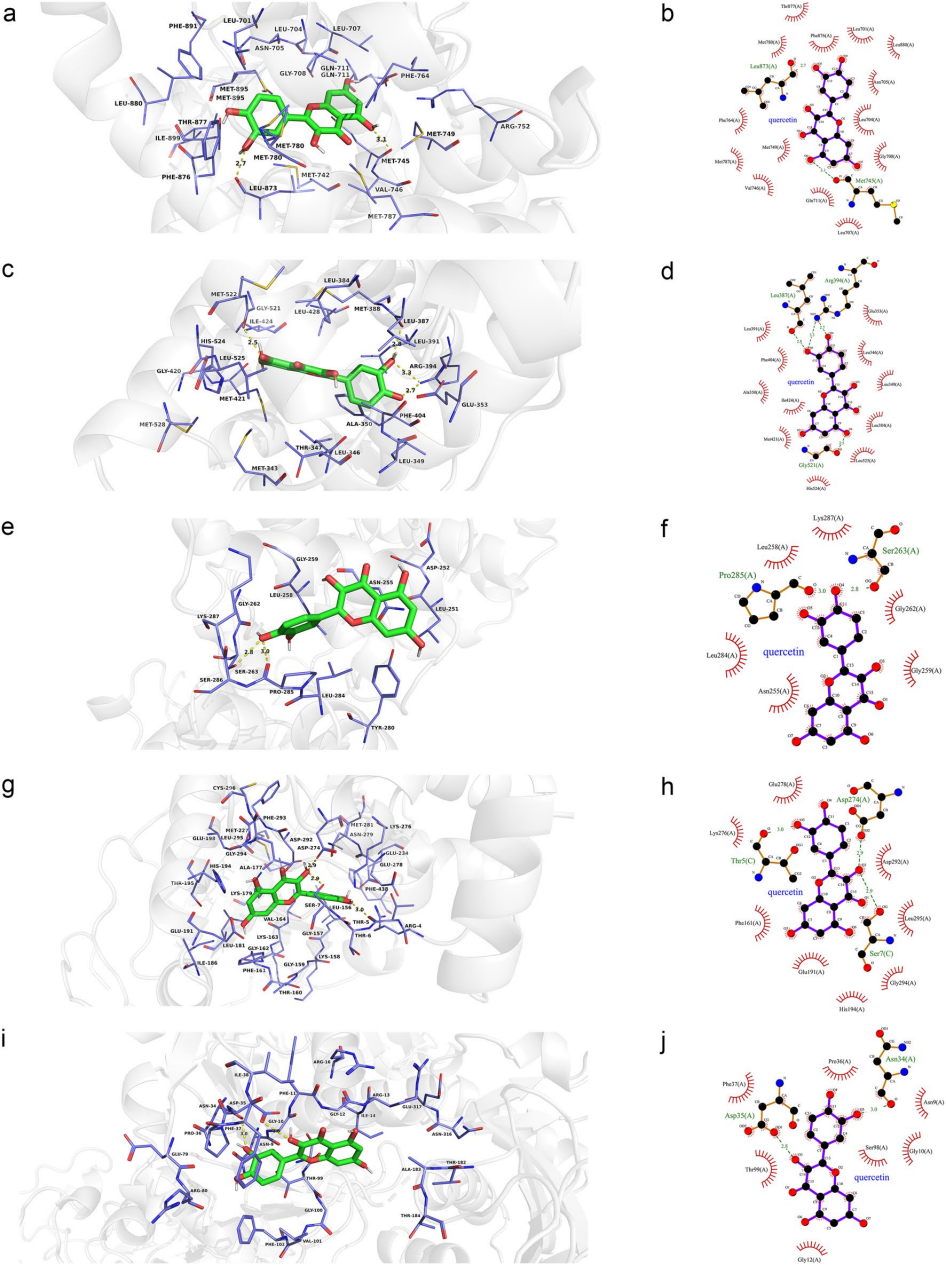
Molecular models of the binding of quercetin from LB to the predicted targets (**a**, **b**) AR, (**c**, **d**) ESR1, (**e**, **f**) MAPK3, (**g**, **h**) AKT1 and (**i**, **j**) GAPDH shown as 3D diagrams and 2D diagrams.

As shown in Fig. 12a,b, quercetin was observed to interact with AR via Thr877, Met780, Phe876, Leu701, Leu880, Asn705, Phe764, Leu704, Met 749, Met787, Gly708, Val746, Gln711, Leu707 and form two hydrogen bonds with Leu873 (2.7 Å) and Met745 (3.1 Å). According to the analysis results shown in Fig. 12c,d, quercetin forms hydrophobic interactions with eleven residues in ESR1 (Leu391, Phe404, Ala350, Ile424, Met421, His524, Leu525, Leu384, Leu349, Leu346 and Glu353) and four hydrogen bonds (quercetin_O5_: Leu387_O_ (2.8 Å), Arg394_NH2_ (3.3 Å), quercetin_O4_: Arg394_NH2_ (2.7 Å), quercetin_O6_: Gly521_O_ (2.5 Å)). Figure 12e,f shows that quercetin was predicted to interact with MAPK3 via Lys287, Leu258, Leu284, Asn255, Gly259 and Gly262, and formed two hydrogen bonds with Pro285 (3.0 Å) and Ser263 (2.8 Å). In addition, the action modes of quercetin and AKT1 are shown in Fig. 12g,h. Quercetin binds to a pocket in AKT1, composing of Glu278, Lys276, Phe161, Glu191, His194, Gly294, Leu295 and Asp292. Three hydrogen bonds, quercetin_O3_: Asp274_OD2_ (2.9 Å) and Ser7_OG_ (2.9 Å) and quercetin_O5_: Thr5_O_ (3.0 Å), further enhance the interactions between the ligand and the AKT1 protein. As shown in Fig. 12i,j, quercetin was predicted to interact with GAPDH via Pro36, Phe37, Thr99, Gly12, Ser98, Gly10, Asn9, forming two H-bonds with the residues Asp35 (2.8 Å) and Asn34 (3.0 Å).

**Figure 13:**
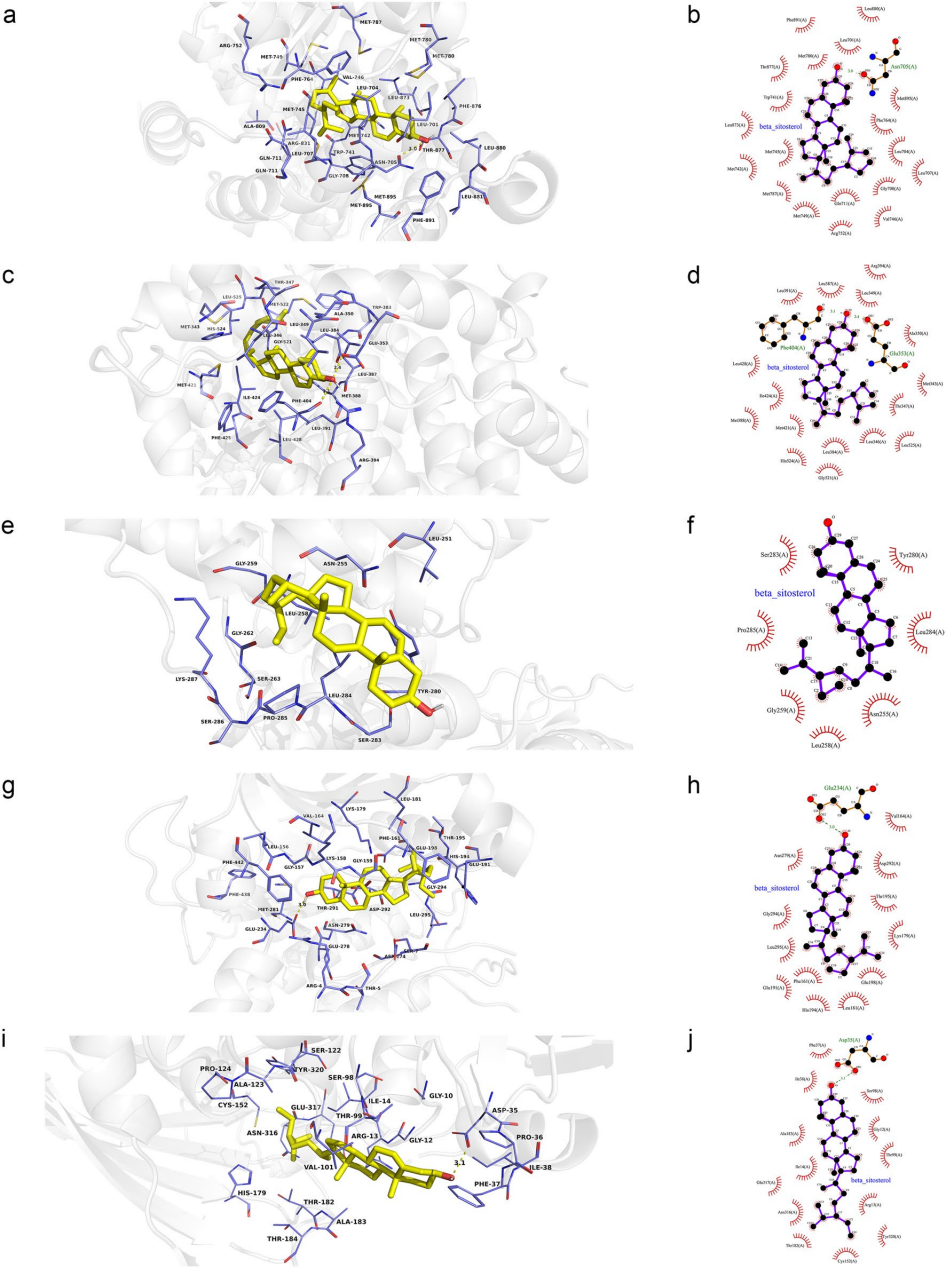
Molecular models of the binding of beta-sitosterol from MO and LB to the predicted targets (**a**, **b**) AR, (**c**, **d**) ESR1, (**e**, **f**) MAPK3, (**g**, **h**) AKT1 and (**i**, **j**) GAPDH shown as 3D diagrams and 2D diagrams.

According to Fig. 13a,b, beta-sitosterol was observed to form hydrophobic interactions with nineteen residues in AR (Phe891, Leu880, Leu701, Met780, Thr877, Trp741, Leu873, Met745, Met742, Met787, Met749, Arg752, Gln711, Val746, Gly708, Leu707, Leu704, Phe764 and Met895) and a hydrogen bond (beta-sitosterol_O_: Asn705_OD1_ (3.0 Å)). As shown in Fig. 13c,d, beta-sitosterol was observed to interact with ESR1 via Arg394, Leu387, Leu391, Leu349, Leu428, Ile424, Met388, Met421, His524, Gly521, Leu384, Leu346, Leu525, Thr347, Met343 and Ala350, forming two H-bonds with the residues Phe404 (3.1 Å) and Glu353 (2.4 Å). Figure 13e,f showed that beta-sitosterol could bind to MAPK3 by forming hydrophobic interactions with the surrounding residues (Ser283, Pro285, Gly259, Leu258, Asn255, Leu284 and Tyr280). Moreover, beta-sitosterol was predicted to interact with AKT1 via Asn279, Gly294, Leu295, Glu191, Phe161, His194, Leu181, Glu198, Lys179, Thr195, Asp292 and Val164 and formed a hydrogen bond with the residue Glu234 (3.0 Å) (Fig. 13g,h). Besides, beta-sitosterol was observed to form hydrophobic interactions with thirteen residues in GAPDH (Phe37, Ile38, Ala183, Ile14, Glu317, Asn316, Thr182, Cys152, Tyr320, Arg13, Thr99, Gly12 and Ser98), and formed a hydrogen bond with the residue Asp35 (3.1 Å) (Fig. 13i,j).

**Figure 14:**
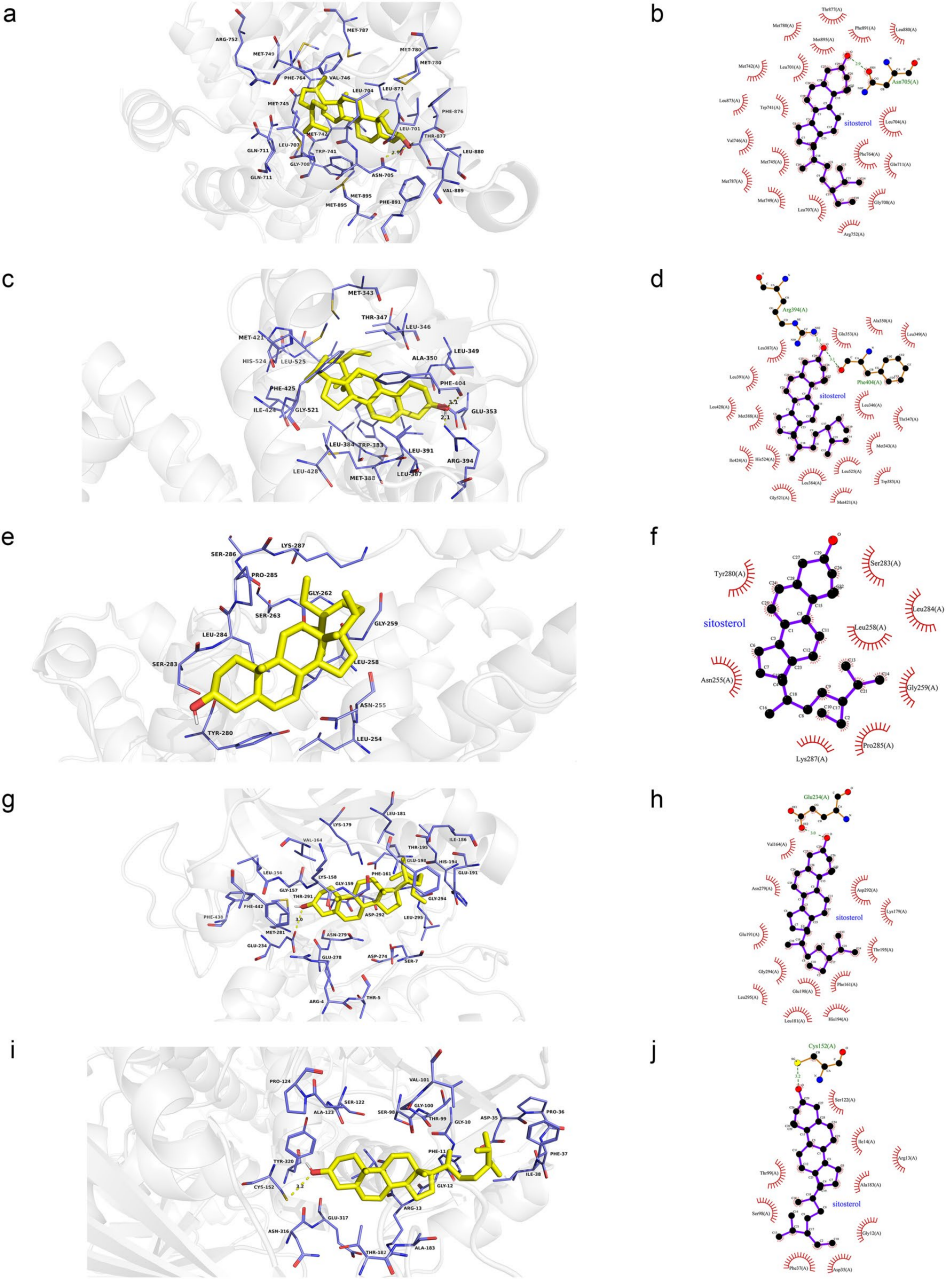
Molecular models of the binding of sitosterol from MO and LB to the predicted targets (**a**, **b**) AR, (**c**, **d**) ESR1, (**e**, **f**) MAPK3, (**g**, **h**) AKT1 and (**i**, **j**) GAPDH shown as 3D diagrams and 2D diagrams.

The action modes of sitosterol and AR are shown in Fig. 14a,b. Sitosterol bound to a pocket in AR, composing of Thr877, Met780, Met895, Phe891, Leu880, Met742, Leu701, Leu873, Trp741, Val746, Met745, Met787, Met749, Leu707, Arg752, Gly708, Gln711, Phe764, Leu704 and a hydrogen bond, Asn705 (2.9 Å). As shown in Fig. 14c,d, sitosterol was predicted to interact with ESR1 via Leu387, Leu391, Leu428, Met388, Ile424, His524, Gly521, Leu384, Met421, Leu525, Trp383, Met343, Thr347, Leu346, Glu353, Ala350, Leu349, forming two hydrogen bonds with Arg394 (2.1 Å) and Phe404 (3.1 Å). According to the analysis results shown in Fig. 14e,f, sitosterol was observed to form hydrophobic interactions with eight residues in MAPK3 (Tyr280, Asn255, Lys287, Pro285, Gly259, Leu258, Leu284 and Ser283). Sitosterol could bind to AKT1 by forming hydrophobic interactions with the surrounding residues Val164, Asn279, Glu191, Gly294, Leu295, Leu181, Glu198, His194, Phe161, Thr195, Lys179, Asp292 and a H-bond with Glu234 (3.0 Å) (Fig. 14g,h). Moreover, sitosterol was observed to GAPDH by forming hydrophobic interactions with the surrounding residues Ser122, Ile14, Arg13, Ala183, Gly12, Asp35, Phe37, Ser98, Thr99 and a hydrogen bond with Cys152 (3.2 Å) (Fig. 14i,j).

## Discussion

With the changes in people’s living habits, environmental pollution, and psychological factors, the incidence rate of infertility continues to rise, where male infertility accounts for 50% of the cases^25^. As one of the most common types of male infertility, OA has a complicated mechanism of action, thus the current treatment methods and drugs have some limitations^26^. So far, the treatment of OA has mainly focused on hormones, anti-infection, surgery, and ART^27^. However, the existing therapeutic drugs and surgical methods could not fundamentally improve the sperm quality of patients with OA. In addition, surgical treatment has brought economic and psychological pressure to infertile couples. Under the current influence of COVID-19 sweeping the globe, researchers have found that the COVID-19 virus can damage the male reproductive system^5–8^. Therefore, it is worth to be further explored to improve sperm quality of OA patients, and develop new drugs against OA.

**Figure 15:**
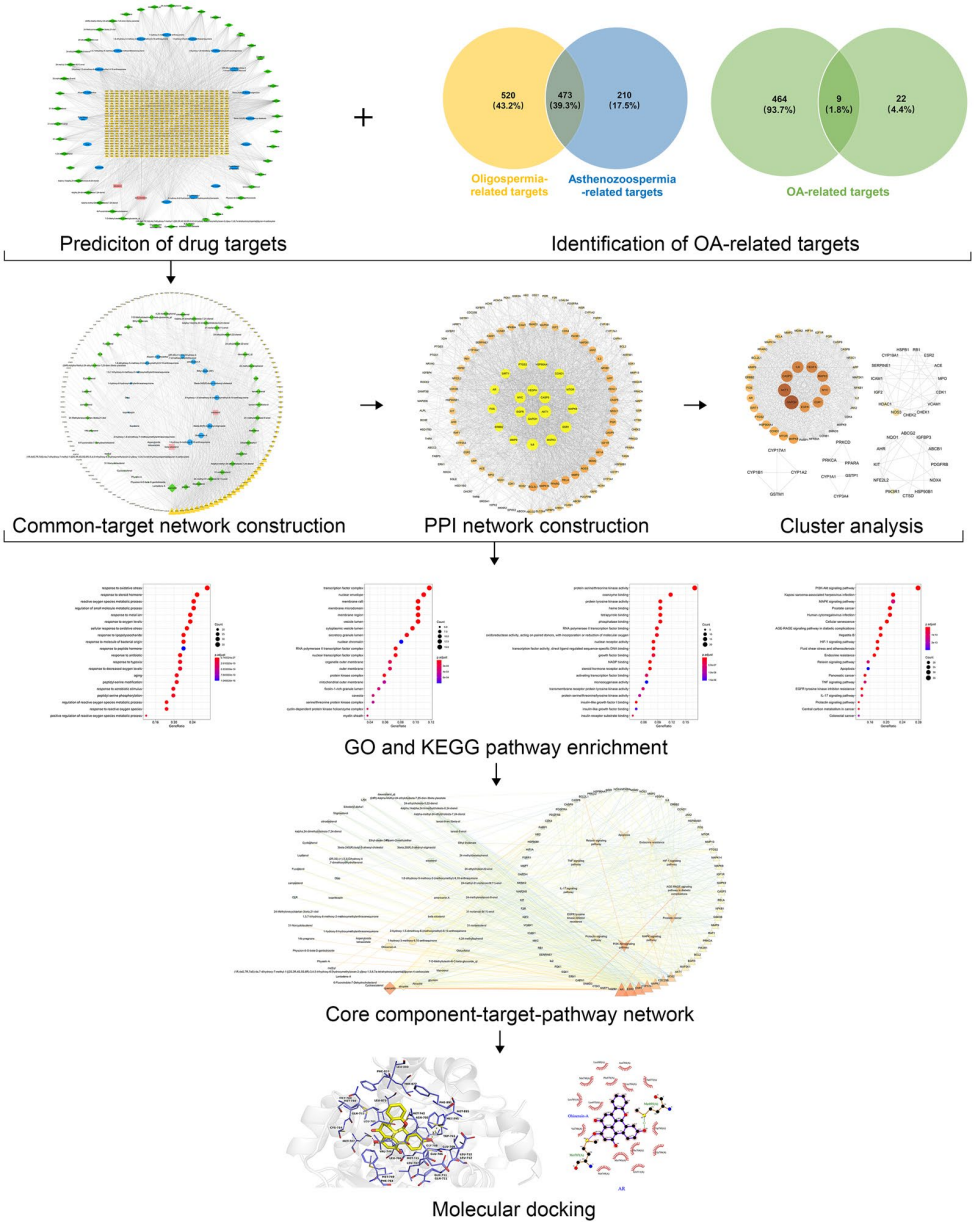
The experimental flow of this study. *OA* oligoasthenozoospermia, *PPI* protein–protein interaction, *GO* gene ontology, *KEGG* Kyoto Encyclopedia of Genes and Genomes.

Here, we adopted a comprehensive method integrated network pharmacology and molecular docking to reveal the bioactive components and potential targets of MOLBCH against OA. The experimental flow of this study was shown in Fig. 15. In our study, for the first time, Ohioensin-A, quercetin, beta-sitosterol and sitosterol were found to be the main bioactive ingredients of MOLBCH against OA. Specifically, the cytotoxic activity of Ohioensin-A has good effects against various cancer cell lines, including murine leukemia cell line and breast cancer cell line^20^. Ohioensin could reduce the TNF-α-induced production of intracellular reactive oxygen species (ROS) and phosphorylation of AKT in vascular smooth muscle cells (VSMCs)^28^. In previous study, quercetin was confirmed to indirectly affect the stimulation of the sex organs, both at the cellular and organ levels^21^, and showed outstanding beneficial effects on the serum total testosterone^22^. The supplement of quercetin could decrease the expressions of AKT, AR, cell proliferative and anti-apoptotic proteins on prostate cancer in the in vivo model^29^. The stimulation of cell proliferation by quercetin is proved to be mediated by ESR1^30^. In a previous study, researchers found quercetin could elicit apoptosis through an ESR1-dependent mechanism in cancer cell lines^31^. Quercetin has a protective effect against chronic prostatitis in rat model through NF-κB and MAPK signaling pathways^32^, and could attenuate cell migration and invasion by suppressing the protein levels of p-AKT1, MMP-2, and MMP-9 in HCCLM3 cells^33^. Beta-sitosterol and sitosterol are natural occurring phytosterols with steroidal moiety, which could inhibit tumor growth, modulates immune response, and has antioxidant capacity. Beta-sitosterol is regarded as a potential chemo preventive agent for treating a variety of cancer, including prostatic carcinoma and breast cancer^23^. It has been reported that beta-sitosterol could inhibit the growth and migration of prostate cancer cell and slow the growth of prostate tumors in mice. Its mechanism of action could be involved in AR^34^. Incorporation of beta-sitosterol into the cell membrane could increase the resistance to OS and lipid peroxidation via ESR1-mediated PI3K/GSK3β signaling^35^. Beta-sitosterol could increase the tyrosine phosphorylation of IRS-1, serine phosphorylation of AKT, threonine phosphorylation of AKT and threonine phosphorylation of Akt substrate of 160 KD in the adipose tissue of type-2 diabetic rat^36^.

In addition, we found that the core potential targets of MOLBCH on OA were AR, ESR1, MAPK3, AKT1 and GAPDH. AR is essential for the development and maintenance of the male phenotype and spermatogenesis^37,38^. Previous studies demonstrated AR might work through testicular Sertoli and peri-tubular myoid cells, maintaining spermatogonia numbers, blood-testis barrier integrity, completion of meiosis, adhesion of spermatids and spermiation^39,40^. ESR1 could regulate expression of genes during the process of spermiogenesis, and has been implicated in male infertility^41–43^. Besides, ESR1 was found to be associated with testicular germ cell cancer, which usually occurs in young men^44^. The MAPKs has been linked to the disturbances in spermatogenesis and dysfunction of germ cells and Sertoli cells, resulting in reduced semen quality and male reproductive dysfunction^45^. In human, MAPK3 may play a crucial role in cell cycle progression and apoptosis^46^. AKT1 is considered as the moderator of cellular growth, survival, metabolism and proliferation^47^. AKT1 could also suppress radiation-induced germ cell apoptosis in vivo^48^ and enhance the effects of thyroid hormone on postnatal testis development^49^. In the testis, GAPDH is of particular importance for spermatogenesis, and could reduce sperm motility induced by male infertility^50^. Besides, EGFR, ESR2, MYC, CASP3, VEGFA, etc. are also important during the process of MOLBCH against OA. EGFR is located in the head and middle of the sperm, and participated in the acrosome reaction and the polymerization reaction of actin in the process of sperm capacitation^51,52^. ESR2 regulates the expression of genes related to germ cell apoptosis and spermatization^41^. c-MYC is an immediate growth early response gene, and might play a key role in cell proliferation and tumorigenesis^53,54^. The increasing expression of CASP3 in the testis may cause germ cell apoptosis in the seminiferous tubules^55^. VEGFA is related to male reproductive function and maintenance of spermatogonial stem cells^56^.

Furthermore, 12 significant pathways were related to apoptosis, male reproductive functions, and OS in the molecular mechanism of MOLBCH against OA. In particular, the most represented signaling pathways are PI3K-Akt signaling pathway, Prostate cancer, and AGE-RAGE signaling pathway in diabetic complications. The PI3K/Akt signaling pathway plays an essential role in inhibiting cell apoptosis and promoting the survival of male infertility^57^. It also perturbs the intracellular redox equilibration by raising ROS^58^. The aberrant activation of PI3K-Akt signaling pathway may contribute to increase cell invasiveness and facilitate prostate cancer progression^59^. Prostate cancer is the second leading cause of cancer death in men, and key determinants of its cellular phenotype include carcinogen defense (GSTP1), growth factor signaling pathways (NKX3.1, PTEN and p27) and AR^60^. Activation of AGE/RAGE signaling pathway promotes ROS production, which can induce lipid peroxidation and eventually sperm DNA damage^61–64^.

Besides, MAPK signaling pathway, IL-17 signaling pathway, TNF signaling pathway, Endocrine resistance, Relaxin signaling pathway, EGFR tyrosine kinase inhibitor resistance, Prolactin signaling pathway, HIF-1 signaling pathway, also play an important role in the process of MOLBCH on OA. The MAPK signaling pathway participates in many stages of germ cell development, including spermatogenesis, germ cell cycle progression, germ cell apoptosis, acquisition of motility in the epididymis, sperm capacitation and acrosome reaction before the fertilization of oocytes^65,66^. The aberrant IL-17 signaling pathway is of great importance to maintain the testicular immune, including cell immunity, mucosal immunity and cytokines^67–69^. TNF family is regarded to stimulate NF-κB, and further acts an effect on varicocele-mediated pathogenesis^70^. Estrogens may be involved in the pathophysiology of varicocele-associated male infertility^71^. In male mice, disruption of the relaxin gene results in the delayed reproductive tract development and arrested sperm maturation^72^. EGFR is a tyrosine kinase related to the regulation of cellular homeostasis. Mutations in the EGFR gene and protein overexpression could activate downstream pathways associated with cancer^73^. Prolactin receptor deficient models built in a previous study showed neuroendocrine and reproductive abnormalities for male rodents^74^. Hypoxia-Inducible Factor (HIF)-1 plays an integral role in responding to low oxygen concentrations or hypoxia in human^75^. Therefore, we believe that MOLBCH could improve male reproductive functions, decrease apoptosis and OS in the treatment of OA, which might further clarify the pathological mechanism of OA.

In summary, we conducted a comprehensive approach integrated network pharmacology and molecular docking to demonstrate the key bioactive components and core potential targets of MOLBCH against OA. We found that MOLCH could alleviate apoptosis, promote male reproductive function, and reduce OS in the treatment of OA. The key bioactive components are Ohioensin-A, quercetin, beta-sitosterol and sitosterol. The core potential targets are AR, ESR1, MAPK3, AKT1 and GAPDH. The most representative pathways are PI3K/Akt signaling pathway, prostate cancer, and AGE-RAGE signaling pathway in diabetic complications. In order to further verify the results of network pharmacology, molecular docking was employed to confirm the strong binding interaction between the key bioactive components and core potential targets. This study provides deeper insights into the pathogenesis of OA and can be helpful to design new drugs and develop new therapeutic instructions to treat OA.

## Methods

### Data collection and processing

#### Components of MOLBCH

TCMSP (http://tcmspw.com/tcmsp.php, updated on May 31, 2014)^76^ and TCMID (http://119.3.41.228:8000/tcmid/, updated on Oct. 24, 2017)^77^ were used to obtain the components of MOLBCH (Supplementary Table S1).

#### Bioactive components of MOLBCH

The adsorption, distribution, metabolism, and excretion (ADME)-related models, integrating oral bioavailability (OB) and drug-likeness (DL), were used to filter the bioactive components of MOLBCH. Oral bioavailability could represent the relative amount of orally administered drug that reaches the blood circulation^78^. Drug-likeliness was used to describe and optimize pharmacokinetic and pharmaceutical properties^79^. According to the published literature^16,19,80,81^, the compounds that meet the requirements of both OB ≥ 30% and DL ≥ 0.18, were used to identify the bioactive components of MOLBCH (Supplementary Table S2).

#### MOLBCH targets

The structure information of the bioactive components of MOLBCH was obtained from PubChem (https://pubchem.ncbi.nlm.nih.gov/, updated on Mar. 31, 2020)^82^ and ALOGPS2.1 (http://www.vcclab.org/lab/alogps/, updated on Feb. 14, 2020). The structure information covered molecular structures, canonical smiles, and their “sdf” files. Then, the targets of bioactive components of MOLBCH were obtained through Swiss Target Prediction (http://www.swisstargetprediction.ch/)^83^ and SEA (http://sea.bkslab.org/)^84^, with the conditions set as “Homo sapiens” and probability value > 0. In addition, we supplemented the prediction targets of MOLBCH from TCMSP and Drugbank (https://go.drugbank.com/) databases. The names of MOLBCH targets were standardized by UniProtKB (https://www.uniprot.org/)^85^ (Supplementary Table S3).

#### OA-related targets

The OA-related targets were obtained by five different databases, including DisGeNET database (https://www.disgenet.org/, updated on May 13, 2019)^86^, Comparative Toxicogenomics Database (CTD, http://ctdbase.org/, updated on Feb. 4, 2020)^87^, Online Mendelian Inheritance in Man (OMIM, http://omim.org/, updated on Feb. 14, 2020)^88^, GeneCards (https://www.genecards.org/, updated on Mar. 11, 2020)^89^ and the National Centre for Biotechnology Information Gene (NCBI Gene, https://www.ncbi.nlm.nih.gov/gene/, updated on May. 4, 2019)^90^. Taking advantage of the different characteristics of each database, we selected “oligoasthenozoospermia”, “oligospermia”, “oligozoospermia”, “asthenospermia” and “asthenozoospermia” as the keywords and criteria to search related targets. We integrated duplicated targets of “oligospermia” and “oligozoospermia”, and defined the obtained targets as the oligospermia-related targets. Similarly, the same targets of “asthenospermia” and “asthenozoospermia” were incorporated, and defined the obtained targets as the asthenozoospermia-related targets. Afterwards, we took the intersection of the oligospermia-related targets and the asthenozoospermia-related targets, and the targets searched by “oligoasthenozoospermia” were added to get the OA-related targets. Finally, we standardized the names of OA-related targets using UniProtKB (Supplementary Table S4).

### Network construction

#### MOLBCH-OA common-target network

The MOLBCH-OA common target network was constructed by the common targets of MOLBCH and OA Cytoscape (http://www.cytoscape.org, version 3.7.2)^91^ (Supplementary Table S5). Network Analyzer^92^, a plugin of Cytoscape, was used for calculating the degree value of the network, aiming to find the underlying components and targets of MOLBCH on OA (Supplementary Table S6).

#### PPI network and evaluation

The STRING database v11.0 (http://string-db.org)^93^ was utilized to get the protein–protein interaction (PPI) information. The confidence score was set to 0.4 or higher. The PPI information were exported in TSV format, and then visualized by Cytoscape. CytoNCA, a plugin of Cytoscape, was used to evaluate the PPI network, including Betweenness (BC), Closeness (CC), Eigenvector (EC), Local Average Connectivity-based method (LAC), Network (NC), Subgraph (SC), Information (IC)^94^. First, the data was screened by using the screening criteria of ‘DC ≥ 2 × median DC’. Then, the potential targets were obtained by the screening criteria of ‘DC, BC, EC, CC, LAC, and NC greater than or equal to their median’^95^ (Supplementary Table S7).

#### Cluster analysis

MCODE^96^, a cluster analysis algorithm in Cytoscape, was performed to analysis the sub-region of PPI network. The same or similar targets were treated as clusters to further explore the underly information the PPI network^97^ (Supplementary Table S8). The conditions were set as node score cutoff = 0.2, K-core = 2, and degree of cutoff = 2.

### GO and KEGG pathway enrichment analyses

Gene Ontology (GO) knowledgebase (http://geneontology.org/), Kyoto Encyclopedia of Genes and Genomes (KEGG) pathway enrichment analyses (https://www.genome.jp/kegg/)^60,98,99^ were conducted by R 4.0.0 software with the Bioconductor package to evaluated the enrichment functions and pathways of the PPI network and clusters (Supplementary Tables S9–S10). The figures of core targets in KEGG signaling pathways are from Kanehisa laboratories (www.kegg.jp/kegg/kegg1.html)^100–102^.

### Molecular docking

Ohioensin-A, quercetin, beta-sitosterol and sitosterol were used as ligands, and AR, ESR1, MAPK3, AKT1 and GAPDH were used as protein receptors. PubChem database (https://pubchem.ncbi.nlm.nih.gov/) was used to download the two-dimensional (2D) structures of Ohioensin-A, quercetin, beta-sitosterol and sitosterol. The 2D structure was processed and transformed into PDB format through Chem3D, and they were saved in PDBQT format as docking ligands in AutoDock Tools 1.5.6 software. The X-ray crystal structures of the targets (http://www.rcsb.org/)^103^, including AR (PDB ID: 2Q7K), ESR1 (PDB ID:4PXM), MAPK3 (PDB ID:6GES), AKT1 (PDB ID:3QKK), and GAPDH (PDB ID:6ADE), were obtained from the Protein Data Bank (PDB). Subsequently, PyMOL 2.4 (https://pymol.org/2/)^104^ was applied to remove water molecules and pro-ligand small molecules. The protein receptor files were processed and then converted to pdbqt format using AutoDock Tools 1.5.6. Each grid box was centered on ligand. Finally, molecular docking calculations were performed using Autodock Vina 1.1.2^105,106^. The conformation with the best affinity was selected as the final docking conformation. The docking results were visualized and displayed as 3D diagrams and 2D diagrams by using PyMOL 2.4 and ligplus.

## Supplementary information

Supplementary information.

Supplementary Table S1.

Supplementary Table S2.

Supplementary Table S3.

Supplementary Table S4.

Supplementary Table S5.

Supplementary Table S6.

Supplementary Table S7.

Supplementary Table S8.

Supplementary Table S9.

Supplementary Table S10.

## Data Availability

The data generated or analyzed during this study are included in the Supplementary Source files.
