# Supplementary material for: Network pharmacology integrated molecular docking reveals the bioactive components and potential targets of Morinda officinalis–Lycium barbarum coupled-herbs against oligoasthenozoospermia
Source: Sci Rep. 2021 Jan 26;11:2220. doi: 10.1038/s41598-020-80780-6 (PMC7838196; doi:10.1038/s41598-020-80780-6)
Supplement: Supplementary file 1 — Supplementary information. [file 41598_2020_80780_MOESM1_ESM.docx]

***Supplementary Information***

**Network pharmacology integrated molecular docking reveals the bioactive components and potential targets of *Morinda officinalis*-*Lycium barbarum* coupled-herbs against oligoasthenozoospermia**

Xue Bai^1^, Yibo Tang^2^, Qiang Li^1^, Yafei Chen^2^, Dan Liu^1^, Guimin Liu^1^, Xiaolei Fan^2^, Ru Ma^1^, Shuyan Wang^2^, Lingru Li^3^, Kailin Zhou^4^, Yanfei Zheng^3, *^, Zhenquan Liu^1, 3, *^

^1^School of Chinese Materia Medica, Beijing University of Chinese Medicine, Beijing, 100029, China

^2^School of Traditional Chinese Medicine, Beijing University of Chinese Medicine, Beijing, 100029, China

^3^National Institute of TCM Constitution and Preventive Medicine, Beijing University of Chinese Medicine, Beijing, 100029, China

^4^School of Humanities, Beijing University of Chinese Medicine, Beijing, 100029, China

*email: yanfei_z@163.com; lzqbzy@sina.com

**Supplementary Tables**

**Supplementary Table S1** Components of each herb in MOLBCH.

**Supplementary Table S2** Bioactive components of each herb in MOLBCH.

**Supplementary Table S3** Potential targets of bioactive components in MOLBCH.

**Supplementary Table S4** Known OA-related targets.

**Supplementary Table S5** 136 common targets between MOLBCH and OA.

**Supplementary Table S6** Analysis of the underlying components of MOLBCH in treating OA.

**Supplementary Table S7** PPI Network centrality analysis and evaluation.

**Supplementary Table S8** The Clusters of the PPI network.

**Supplementary Table S9** GO and KEGG pathway enrichment analyses for the PPI network.

**Supplementary Table S10** GO and KEGG pathway enrichment analyses for cluster 1.
